# Bionanocomposite Four-Channel Biosensor for Rapid and Convenient Monitoring of Glucose, Lactate, Ethanol and Starch

**DOI:** 10.3390/gels11050355

**Published:** 2025-05-12

**Authors:** Anna Kharkova, Lyubov Kuznetsova, Roman Perchikov, Maria Gertsen, Pavel Melnikov, Nikolay Zaitsev, Jun Zhang, Vyacheslav Arlyapov

**Affiliations:** 1 The Research Center «BioChemTech», Tula State University, 300012 Tula, Russia; anyuta_zaytseva@mail.ru (A.K.); l.s.latunina@gmail.com (L.K.); perchikov_roma@mail.ru (R.P.); 2 Laboratory of Soil Chemistry and Ecology, Tula State Lev Tolstoy Pedagogical University, 300026 Tula, Russia; mani.gertsen@gmail.com; 3 M.V. Lomonosov Institute of Fine Chemical Technologies, MIREA—Russian Technological University, 119571 Moscow, Russia; 4 Econics-Expert Ltd., Akademika Bakuleva St., 6, 117513 Moscow, Russia; 5 National Key Laboratory of Urban and Rural Water Resources and Water Environment, School of Environment, Harbin Institute of Technology, Harbin 150090, China

**Keywords:** redox-active gel, BSA g, phenazines, food quality assessment

## Abstract

A biosensor for the determination of glucose, lactate, ethanol and starch in beverages has been developed using enzymes immobilized by a redox-active gel on a screen-printed electrode. A significant improvement proposed for multichannel biosensors, overcoming stability and sensitivity issues by covalently binding phenazine mediators to a biocompatible protein hydrogel, enhancing the packaging of the enzyme. Glucose oxidase (GOx), alcohol oxidase (AOx) and lactate oxidase (LOx) were used as biological materials, as well as a mixture of GOx with γ-amylase (Am). Redox gels were synthesized from bovine serum albumin (BSA) and phenazine derivatives. It was shown that a neutral red-based redox gel combined with single-walled carbon nanotubes is more promising than other substrates for enzyme immobilization. The lower limit of quantification for glucose, ethanol, lactate and starch using these systems is 0.035 mM, 2.3 mM, 15 mM and 2 mg/L, respectively. Biosensors were used to analyze the content of these substances in alcoholic, kvass and fermentation mass. Statistical analysis of the results showed that the values of glucose, ethanol, lactic acid and starch determined using biosensors and obtained by reference methods differ insignificantly. A set of biosensors developed on the basis of specifically selected enzymes is effective for controlling biotechnological processes and can be used as an alternative to classical analytical methods.

## 1. Introduction

**Figure 1:**
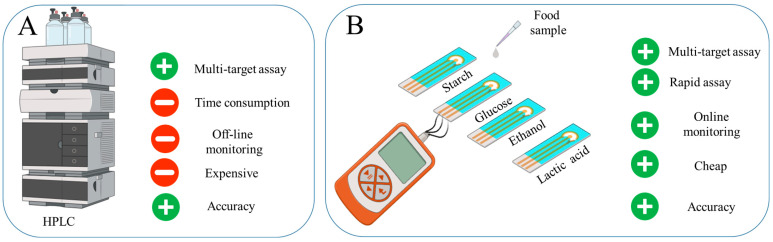
The development of a rapid system for food product evaluation to monitor starch, lactate, ethanol and glucose in food products: (**A**) Advantages of HPLC. (**B**) Developed four-channel biosensor.

Ensuring food safety through simple and rapid screening for contaminants is critical in protecting human health [1,2]. Traditional methods of food testing frequently rely on laboratory procedures (Mass Spectroscopy, High-performance Liquid Chromatography (HPLC), Nuclear Magnetic Resonance, Polymerase Chain Reaction, Enzyme-linked Immunosorbent Assay, Lateral Flow Devices), which can be labor-intensive and may delay the detection of potential hazards, thereby increasing the risk of consumer exposure to harmful substances [3,4,5,6]. Amperometric biosensors are an effective tool for rapid quality monitoring, allowing almost instant detection of small changes [3]. Such devices can be portable and adapted to monitor a variety of substances, making them versatile for quality control [7]. In general, enzyme-based amperometric biosensors provide high efficiency and reliability under conditions where speed and accuracy of analysis are critical [8] (Figure 1).

To date, many electrochemical glucose detection platforms have been developed for disease diagnosis and food quality monitoring. The GOx enzyme often forms the basis of a biosensor for glucose detection, providing high stability to pH and temperature and selectivity [9,10]. Although there are many glucose-oxidase-based sensors and disposable glucose detection devices on the market, portable and durable glucose monitoring devices that provide continuous detection are still in high demand because of low long-term stability [11,12,13,14,15,16]. Amperometric biosensors for ethanol based on the use of the enzyme AOx are widely used as an alternative to standard control methods [17,18]. The main physiological function of the AOx enzyme is methanol oxidation, it also has the ability to oxidize other short-chain alcohols, such as ethanol, propanol and butanol [18]. The study by Stasyuk et al. confirms the effectiveness of using immobilized enzymes with nanoparticles of PtCu and 3,3′,5,5′-tetramethylbenzidine for ethanol analysis in beverages, with an ethanol detection range of 10–150 μM. The system was tested on three wine samples [17]. The development of multiplex biosensors, which can simultaneously detect several analytes in a single sample, has generated significant interest owing to their potential to provide a more comprehensive analysis with greater economic efficiency [19]. A two-channel biosensor for detection of ethanol (0.1–0.9 mM) and glucose (0.01–0.15 mM) in grape must and wine [20] has also been investigated. The biosensor consisted of two electrodes, modified with carbon microfibers, hemin and gold nanoparticles, and each analyte used AOx or GOx enzymes fixed with Nafion membrane.

Lactic acid is a food additive (E270). The level of lactate in food products is an important indicator of the degree of fermentation. The maximum amount of this additive is set for nectars only (5 g/L). For the rest, it is set according to technical documentation [21]. Amperometric enzyme biosensors are being developed using the enzymes lactate dehydrogenase [18,22] or LOx [23,24,25] to determine lactate levels. In the study [22], a chip with several sections for immobilized enzymes was developed and used for multi-control fermentation process and propionate (0–1.5 mM), acetate (0–1.4 mM), D-Lactate (0–2.5 mM), L-Lactate (0–2.0 mM), Formate (0–3.0 mM) and Ethanol (0–1.5 mM) determination. Despite the advantage of simultaneous analysis of a complex sample, the composition of the electrolyte is quite complex: thiamine pyrophosphate, MgCl_2_, flavin adenine dinucleotide disodium salt, phospho(enol)pyruvic acid monopotassium salt, Adenosine 5′-triphosphate disodium salt, acetyl-CoA and potassium ferricyanide.

Starch is used widely in the food industry as a thickener, binder and filler. It is also used in baby and dietary foods and soft drinks. Its content in food is usually regulated by quality and safety standards. The content of starch used as a preservative in food products should not exceed 50 mg/kg [21]. The maximum starch level in baby formula should be no more than 20 g/L [21]. In complementary foods, it should be 50 g/kg or less [21]. Despite the rare development of amperometric biosensors for starch determination, Liu et al. propose a new method that uses a specific enzyme for starch hydrolysis, which allows it to be analyzed amperometrically in the range of 50–3500 mg/L [26]. Thus, research in the field of enzyme-based amperometric biosensors continues to evolve, developing systems that allow analysis of several components simultaneously [27].

Based on recent reviewers [1,2,3,4,5,6,7,28,29], there are two challenges for an enzymes-based biosensor. The first one is that biosensor systems are mainly used for the assessment of a single component [6]. Previously, our research group created laboratory models of multichannel biosensors, but they were characterized by low stability and sensitivity due to the constant leaching of the mediator from near-electrode spaces [30,31]. The second challenge is low long-term stability, and most of the reported investigations are not measured this parameter [6]. In this paper, this problem is proposed to be solved by covalently binding phenazine mediators to a biocompatible protein hydrogel in which the enzyme is packaged. Thus, the aim of this work is to develop an amperometric biosensor that allows continuous analysis of four components: lactate, ethanol, glucose and starch. The analysis is based on the reactions of these components with enzymes, including GOx, LOx, AOx and a mixture of Am and GOx immobilized in a composite material of redox-active gel and carbon nanoparticles. The use of redox polymers in combination with nanoparticles improves electrical conductivity and the kinetics of electron transfer through immobilized enzymes [32]. Biocompatible polymers and nontoxic redox particles improve the stability of enzymes and therefore improve the performance of the biosensor [33].

## 2. Results and Discussion

### 2.1. Design of a Biosensor for Monitoring Glucose, Ethanol, Lactate and Starch

**Figure 2:**
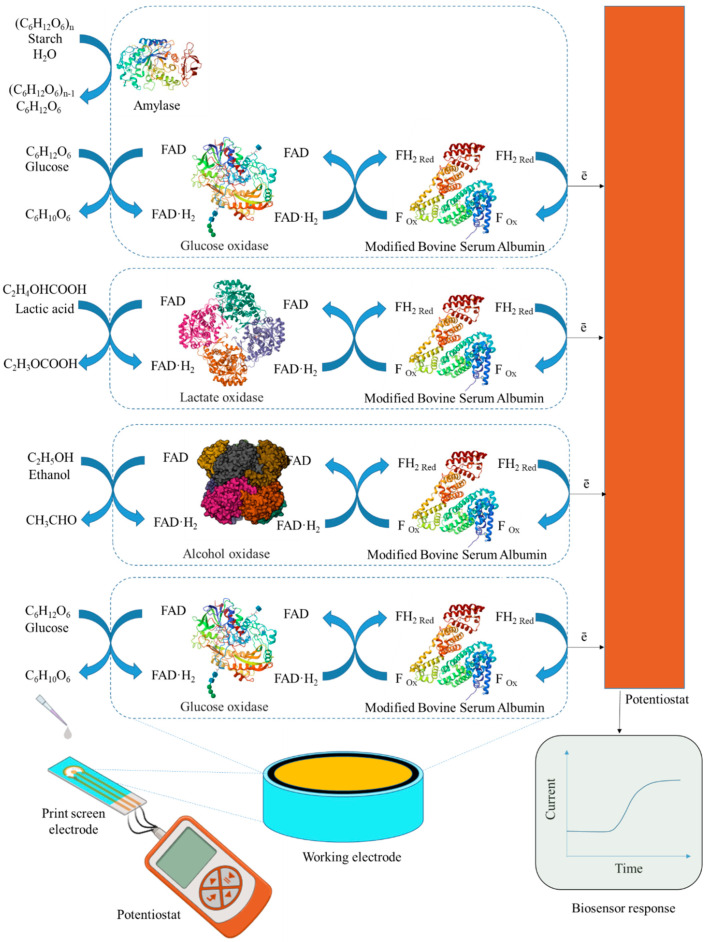
Design of a biosensor and component receptor elements for the analysis of glucose, lactate, starch and ethanol.

In this work, the development of a 4-in-1 biosensor based on enzyme-printed electrodes for the determination of glucose, ethanol, lactate and starch is investigated. Enzymes from the oxidase class were used to create electrodes for the detection of glucose and lactate. GOx was chosen as the primary enzyme for glucose determination, as it has a high specificity for β-D-glucose and catalyzes its oxidation to gluconeolactone. For ethanol detection, printed electrodes were modified with AOx, which is not as specific as GOx but simplifies the process by not requiring the addition of cofactors that cannot be immobilized. The lactate content was determined using printed electrodes modified by LOx. To assess starch levels, a combination of Am and GOx was used in printed electrodes. Am catalyzes the hydrolysis of β-glucose from the non-reducing end of amylose and amylopectin by cleaving α-1,4, α-1,6 and α-1,3 glycosidic bonds. The resulting glucose is then oxidized at the electrode using GOx to gluconolactone. For glucose, lactate and ethanol detection, enzymes are mixed separately into distinct gel preparations, with each formulation applied to individual electrodes for specific analyte detection. GOx with γ-amylase is combined into distinct gel preparations and applied to individual electrodes for starch detection (Figure 2).

To register the analytical signal formed due to the interaction of enzymes with the components being determined, the working electrode was modified by immobilizing enzymes in a composite based on carbon nanotubes and redox-active gel obtained by modifying the BSA gel with electroactive compounds–phenazine derivatives. Redox gels based on these compounds are characterized by rapid electron transfer and electrocatalytic activity, and are, therefore, frequently used to develop electrochemical biosensors [34,35,36,37,38,39]. The introduction of carbon nanotubes into the composite allows the increase in electrical conductivity in the system and retains the enzyme on the electrode surface, which helps to improve the characteristics of the biosensor [40,41]. The presence of functional groups (amino groups) in phenazine and BSA molecules allows the formation of redox-active gel using glutaraldehyde. It should be noted that the successful use of redox-active polymers BSA and neutral red forms a biosensor to assess BOD and toxicity [38] and that BSA and safranin are used to assess the phenolic index [39].

**Figure 3:**
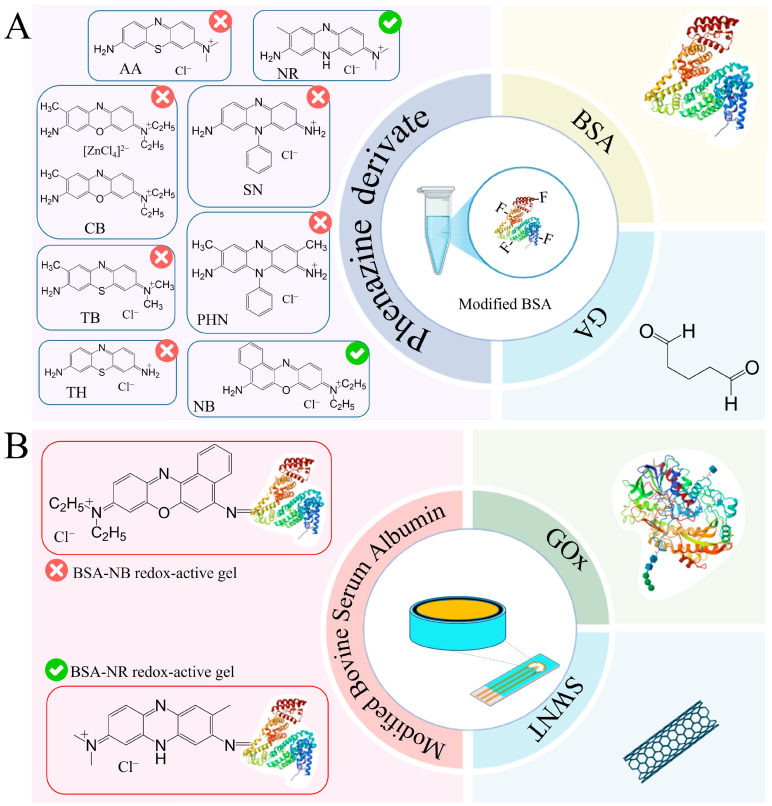

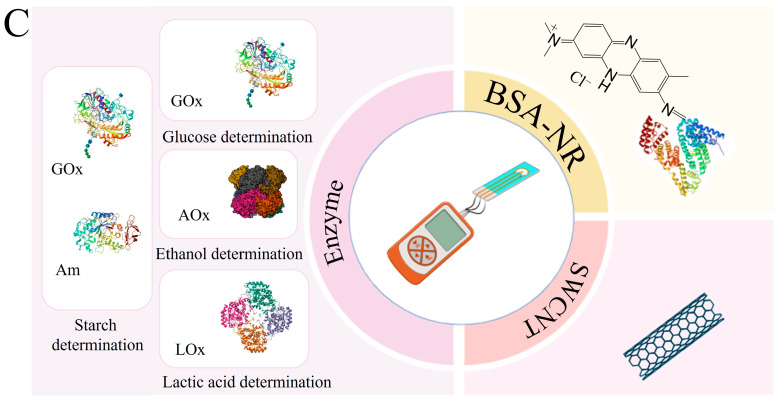
Steps in the development of a 4-in-1 biosensor for food monitoring using bionanocomposites: (**A**) The 1st step is a redox-active gel formation. (**B**) The 2nd step is nanocomposite formation. (**C**) The 3rd step is biosensor formation.

Taking into account the advantages of redox-active polymers based on BSA, phenazine compounds are capable of mediating electron transfer from active enzyme centers to the electrode surface and carbon nanotubes, and a new type of reagent-free biosensor for glucose, lactate, ethanol and starch based on a BSA-neutral red/CNT composite was developed in this work. Each stage of formation is described below and illustrated in Figure 3.

### 2.2. Formation of a Redox-Active Gel

**Figure 4:**
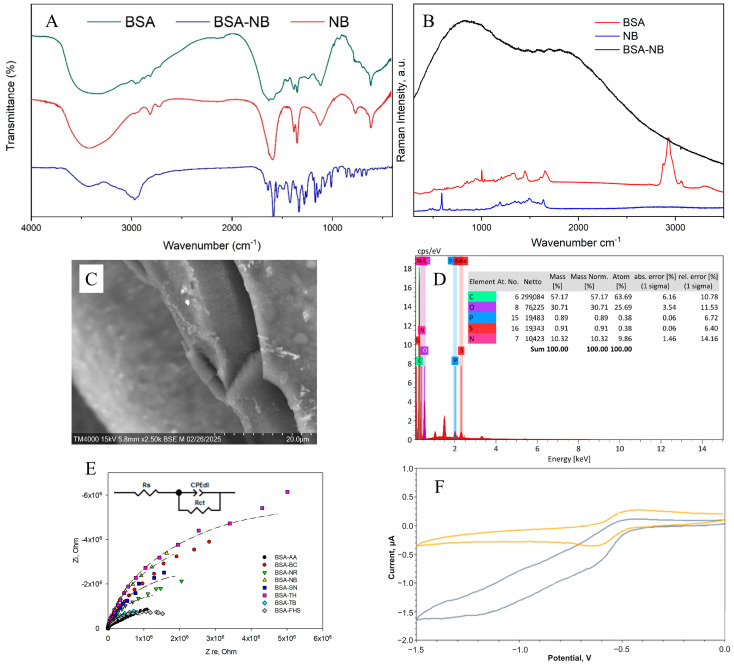
Formation of redox-active gel BSA-NB: (**A**) IR spectra of initial monomers and polymers, (**B**) Raman spectra of initial monomers and polymers, (**C**) SEM of polymer, (**D**) EDX of polymer, (**E**) Impedance spectra, (**F**) Change in cyclic voltammograms before (purple line), and after (orange line) adding glucose into the system.

Figure 4 shows the results of applying all the methods used to analyze the chemical structure of the redox-active gel of BSA-NB. The modification of BSA with electroactive phenazines was confirmed by IR and Raman spectroscopy (Figure S1): an absorption band at 1600 cm^−1^ appears in the IR spectrum of the redox-active polymers, which corresponds to the stretching vibrations of the C=N imine bond of the Schiff base, which is consistent with earlier studies [38,39,40,41,42]; strong fluorescence of the polymer is observed in the Raman spectra, indicating that new gel units have been formed, in contrast to the original pure substances (Figure S2). The change in the surface structure of the developed electrodes was assessed using SEM micrographs. The polymer structure obtained after cross-linking the molecules of phenazine and BSA derivatives with glutaraldehyde is monolithic, contains small pores and is capable of firmly holding enzyme molecules without interfering with the diffusion of substrates and metabolites (Figure S3).

The electrochemical properties of bioelectrodes based on redox-active gel were studied using cyclic voltammetry (Figures S4 and S5) and impedance spectroscopy (Figure S7). Electron transfer in the redox gel occurs through the process of hopping electron transfer between mediator molecules. This process can be limited by either the hopping stage or the step of electron transfer from the mediator to the electrode surface, which is known as the surface reaction at the electrode [43].

To evaluate the electron transfer by redox-active polymers, the constants of heterogeneous electron transfer and the rate constant of interaction between the redox polymer and the GOx enzyme, k_int_, were obtained (Table 1). The Laviron model was used to calculate the rate constants of heterogeneous electron transfer (Text S1, Equation (S1)). The Nicholson-Shine model was used to calculate the rate constant of interaction between the redox gel and GOx (Text S2, Equation (S2)). The obtained data (Figures S4–S6) made it possible to quantitatively measure the rates of the individual stages of electron transfer in biosensors and thus, evaluate the efficiency of interaction between the enzyme, redox site and electrode (Table 1).

The obtained results show that NB and NR can be distinguished as the most effective modifications of BSA for the formation of redox-active polymers based on the interaction rate constants. The average electrolyte resistance Rs was 282 Ohm. The double electric layer of all polymers has a similar roughness αdl, with a low capacity of 0.07–0.18 μF. The lowest charge transfer resistance is observed for redox polymers based on BSA and the mediators neutral red, toluidine blue, phenosafranin and azure, respectively. Based on the obtained data, two mediators can be distinguished: neutral red (by high values of the heterogeneous rate constant, interaction constant and charge transfer resistance) and NB (by high values of the heterogeneous rate constant and interaction constant) for the creation of nanocomposites based on them.

### 2.3. Formation of a Nanocomposite Based on Redox-Active Gel BSA-NR and BSA-NB

**Figure 5:**
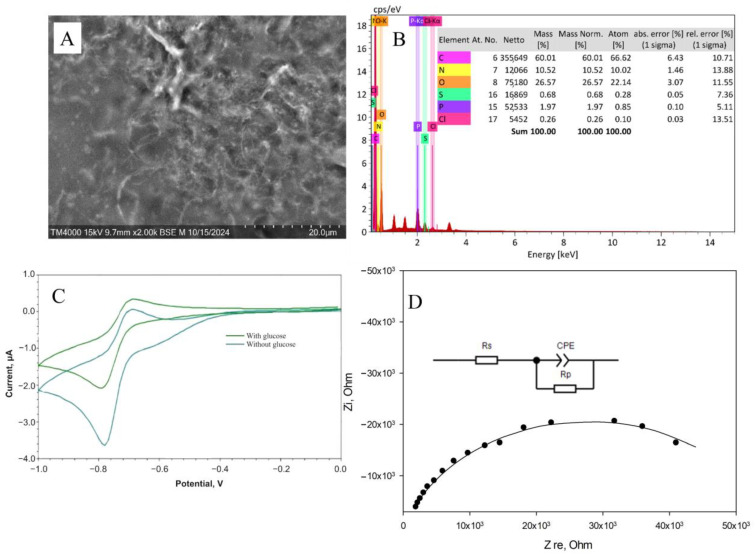
Formation of the BSA-NR-CNT composite: (**A**) SEM image of the gel. (**B**) EDX analysis of the gel. (**C**) Changes in cyclic voltammetry upon the introduction of glucose into the system. (**D**) Impedance spectroscopy.

Carbon nanomaterials, used as electrode surface modifiers, are known for their ability to have a positive effect on conductivity and sensitivity due to the formation of conductive carbon frameworks in the volume of composite material. However, it has been found that in some cases, carbon materials can have an inhibitory effect on enzymes and can lead to a 4–5-fold decrease in activity [44]. The biocompatibility of the gel is crucial in maintaining the native structure and functionality of enzymes embedded within it. A biocompatible gel provides a non-toxic, supportive microenvironment that preserves enzyme activity by preventing denaturation or degradation [45]. This environment can regulate enzymatic functions by allowing proper substrate diffusion and maintaining optimal conditions such as pH and ionic strength [46]. From this perspective, biocompatibility of redox-active gel and composites is extremely important. In the presence of nanotubes, rates of electron transfer to electrodes and interaction of redox-active polymer particles with enzymes were observed (Figure 5 and Figures S6–S10). 

The parameters found are presented in Table 2.

It should be noted that, with the introduction of carbon nanotubes, the rate constant for heterogeneous electron transfer did not change when polymers based on NB were used. When using redox gel based on NR, however, the rate increased by almost 2 times due to the nanotubes’ ability to provide electron transfer between the electroactive regions of the polymer where electron transfer had been kinetically hindered [49]. It should be noted that, in the case of using a composite based on BSA modified with NB, the introduction of nanomaterials reduces the rate of interaction between redox particles and the enzyme. In the case of BSA modification with NR, this rate increases significantly. However, the process of electron transfer to the electrode in this system is inferior to that of other electrochemical systems [47,48]. Nevertheless, the high rate at which the enzyme interacts with redox particles when using a composition based on BSA-NR indicates the promise of its use in analytical systems. When analyzing impedance spectra after introducing nanoparticles into the system, Rct values decrease, indicating faster electron transfer. Therefore, a composition of BSA-NR and carbon nanotubes is used for these enzymes.

The rate constants of interaction of the BSA-NR gel with other enzymes are presented in Table S1. For the first time, the interaction constants for the enzymes AOx, LOx and a mixture (Am + GOx) for nanocomposite materials based on BSA were determined. Moreover, the enzyme GOx has the highest rate constant of interaction in the developed conducting system (17,000 L/mol·s). Efficient bioelectrocatalysis was confirmed by impedance spectroscopy: the Rct value decreased upon the addition of the substrate to the cell, and by amperometry: at a given potential in the system, an analytical signal was generated, the value of which depended on the concentration of the introduced analyte.

### 2.4. Selection of Operating Parameters

**Figure 6:**
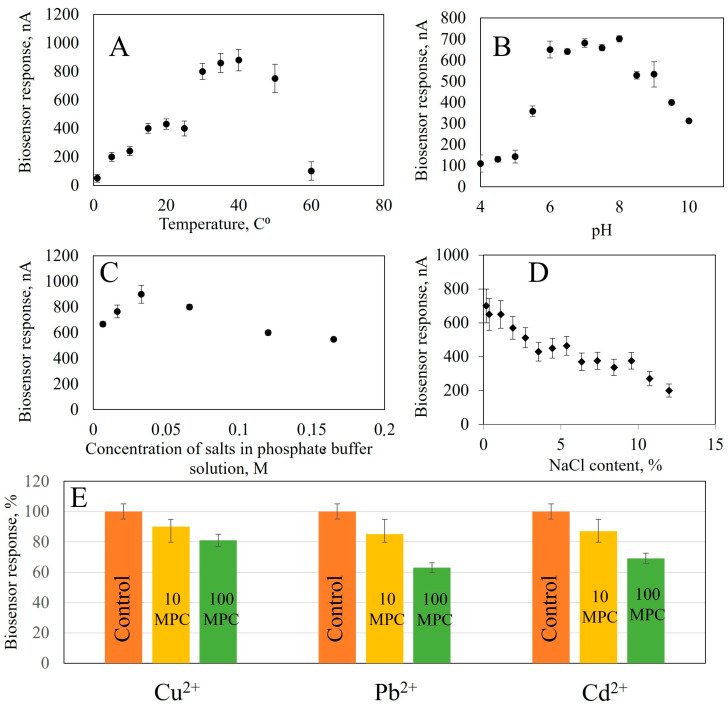
The effect on the analytical signal of the bioreceptor element based on GOx: (**A**) Temperature, (**B**) pH, (**C**) Concentration of buffer solution salts, (**D**) NaCl content, (**E**) Presence of heavy metal ions (control sample without added heavy metals, 10 maximum permissible concentrations (MPC)—concentration exceeding 10 times the permissible values, 100 MPC—concentration exceeding 100 times the permissible values.

In order to create a biosensor for monitoring food product quality, it is necessary to select operating parameters for receptor elements, such as analysis temperature, pH of working electrolyte and concentrations of buffer salt solution. To this end, the biosensor response was recorded as change in current before and after the introduction of a component under investigation (Figure 6). Temperature has a significant impact on enzyme biosensor performance, so changes in biosensor responses were studied when the temperature varied from 1 to 60 °C (for starch up to 70 °C). All measurements were conducted in a thermo-regulated cell. Temperature in the cuvette was adjusted using a measuring probe. Data for other enzymes are presented in Figure S11.

From the obtained dependencies, it follows that when analyzing for glucose content, the maximum response of the biosensor is observed at 50 °C; according to the literary data, the optimal temperature of Aspergillus niger α-glucosidase is in the range from 50 to 65 °C, A. niveus—65 °C, A. nidulans—60 °C and A. niger M1—50 °C [50,51]. The biosensor for ethanol assessment generates a maximum analytical signal at 35 °C. The highest response is achieved in the range of 20–40 °C, but in this temperature range, the stability of the analytical signal decreases: with a change in temperature of 5 °C, the biosensor’s response changes by 10%. Biosensors based on immobilized AOx enzymes have a maximum response at 45 °C [49] and 50 °C [52], respectively. The temperature optimum of an enzyme depends on the source from which it was isolated. AOx isolated from thermophilic yeast O. polymorpha has a temperature optimum that is shifted towards higher temperatures (up to 45 °C), which contrasts sharply with the temperature of AOx from P. pastoris, which is 25 °C [53]. The optimum temperature for lactate determination is in the range of 35–40 °C, which corresponds to the optimum for known models of lactate biosensors 32–34 °C [54], 35 °C [55]. To determine starch in the receptor element, two enzymes are used; the temperature optimum for this receptor element is in the range of 40–60 °C.

The pH of the medium is one of the important factors affecting enzyme activity and, consequently, biosensor sensitivity. In this work, we investigated the biosensor response to pH changes from 4 to 5 using acetate and from 5 to 10 using a sodium-potassium phosphate buffer solution (Figure S12). The experiment revealed that the maximum biosensor response based on GOx was observed in pH range 5.6–7. AOx based biosensors worked most effectively between 5 and pH 9, while LOx were optimal at pH 6–8 and GOx and Am at 5.5–7. All biosensors showed good performance over a wide range of pH, comparable to their natural conditions [49,50,51,52,53,54,55].

The next parameter that influences the analytical signal of the biosensor is the ionic strength which changes the conductivity of the sample and, therefore, the generated current. The influence of ionic strength on the biosensor response was studied by recording the response of enzyme biosensors at different NaCl concentrations (from 0.2 to 12%). NaCl was chosen because it is an indifferent electrolyte with good solubility and can be present in the samples being studied [56]. With increasing salinity in the medium, the biosensor responses decrease. This can be seen in Figure S13. Data obtained show that salinity has a detrimental effect on enzyme biosensor operation. At a salt concentration of 12% in the cell, the response to glucose decreases by 62%, ethanol by 79%, lactate by 71% and starch by 69%. Note that the analytical signal from enzyme biosensors is more stable in the presence of sodium chloride than in analogues. In [57], it is described that the activity of immobilized GOx decreases five times at a concentration of 200 mM NaCl (1.16%), compared to salt-free solutions. In another work, [58], a decrease of nearly 50% in enzyme activity is indicated upon reaching a concentration of NaCl of 0.5 M (3%) while in [59], the reaction of an electrode based on LOx decreased gradually to two-thirds at concentrations between 40 and 50 mM, with an ionic strength of 0,23% and 0.3%. Then, the effect of the ionic strength was eliminated in [60], where a stable sensor was described based on AOx, demonstrating stable responses up to a NaCl concentration of 100 mM (0.6%). After that, a gradual decrease in sensor response was observed. The available data allow us to conclude that obtained nanocomposite materials create a protective environment during enzyme immobilization. This protects them from the action of salts and allows analysis to be carried out in more saturated solutions. This is especially important for the analysis of food products and the quality control of biotechnological processes. The dependence of the biosensor response on the buffer solution salt concentration was studied in the range of 0.0066–0.1650 mol/L at pH 7.0 of a sodium-potassium phosphate buffer (Figure S14). The maximum response was observed for all biosensors at a buffer concentration of 0.033 mol dm^−3^. Therefore, this concentration is optimal for analysis.

Heavy metal ions have the ability to reduce enzyme activity. To study the inhibitory effect of heavy metal compounds, we investigated the dependence of biosensor responses on the presence of Cu^2+^, Cd^2+^ and Pb^2+^ ions in a solution with a concentration range exceeding the maximum permissible concentrations of heavy metals in food raw materials and food products by 10 and 100 times [61]. Monitoring the concentration of these ions is important in biotechnological production, for example in wine production. Large amounts of Cu^2+^ ions can cause oxidative spoilage in wines, leading to clarification of red wines and darkening of whites [62]. Accumulation of Cd^2+^ and Pb^2+^ due to use of contaminated raw materials or during technological processes leads to accumulation in the final product [63]. In the beer industry, trace elements, including heavy metals, make up approximately 0.02% of malt extract [64].

To assess the effect of heavy metal ions on biosensor response, we studied how the biosensor responds to adding substrates in the presence of different concentrations of salts. The concentrations were taken to be exceeding 10 and 100 times the MPC [61]. The data from measurements are presented in Figure S15. The presence of Cu^2+^ ions had the greatest effect on GOx activity, with a decrease in response by a factor of 44% when the concentration was increased by 10-fold. When the concentration exceeded 10, the decrease was between 11 and 13% for all the heavy metals studied, indicating the stability of the GOx enzyme immobilized on a composite material based on carbon nanotubes modified with BSA and NR. Pb^2+^ had the most significant effect on AOx, causing a reduction in activity by 33%. When the MPC is exceeded by 100 times, the responses decrease by 30% for Cu^2+^ ions and by 22% for Cd^2+^. The greatest influence on LOx activity is exerted by Pb^2+^ and Cd^2+^, with an increase of 10-fold in the MPC resulting in a 38% and 33% decrease, respectively. The activity of immobilized Am and GOx is also affected by the presence of these ions, with a decrease of 28%. The results indicate that the enzymes are stable in immobilized nanocomposites, even at concentrations of up to 10 times the MPC.

Thus, for each receptor element, operating parameters were identified. The temperature optima for the operation of the receptor elements were found to be 50 °C for assessing glucose, 35 °C for ethanol, 35–40 °C lactate and 40–60 °C starch; pH of the working electrolyte 7 and the sodium-potassium phosphate concentration buffer 0.033 M for all analytical systems studied.

### 2.5. Main Characteristics of the Biosensor

**Figure 7:**
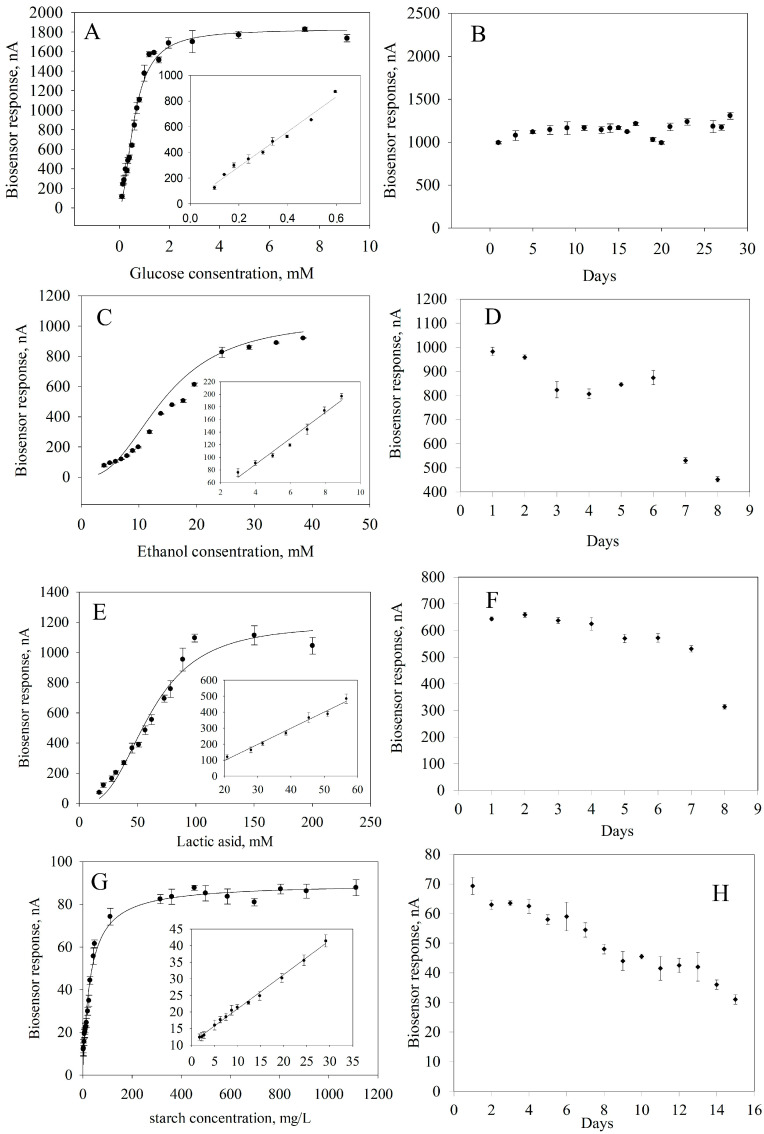
Biosensor performances: (**A**) Calibration curve for glucose determination. (**B**) Long-term stability of GOx. (**C**) Calibration curve for ethanol determination. (**D**) Long-term stability of AOx. (**E**) Calibration curve for lactide acid determination. (**F**) Long-term stability of LOx. (**G**). Calibration curve for starch determination. (**H**) Long-term stability of GOx and Am.

To obtain quantitative information about the content of the analyzed substances in a sample, it is necessary to know the calibration characteristics of a biosensor, that is, the dependence of an analytical signal on the concentration of a determined substrate (Figure 7). These dependencies were approximated using a hyperbolic equation with two parameters because the formation of an analytical response occurs within classical Michaelis–Menten kinetics [65]. From these calibration dependencies, linear regions were identified where the reaction between a substrate and an enzyme follows a first-order mechanism according to the Michaelis–Menten equation.

The main analytical and metrological parameters of a bioreceptor element based on immobilized enzymes in a composite material were compared with those of analogues of glucose, lactic acid, ethanol and starch analyzers (Table 3) [26,66,67,68,69,70,71].

It should be noted that a large number of models of electrochemical biosensors have been developed for the analysis of lactate, glucose, starch and ethanol in the food industry [6]. A modern approach is to simultaneously analyze several analytes when analyzing complex samples. The device allows for determining the content of starch, glucose, lactate and ethanol when they are present in samples of fermented and non-fermented media, liquor and vodka, as well as glucose and molasses, among other industries. Adsorbed ferrocene washes out gradually from the analytical system to assess glucose and lactate levels [67]. Systems for assessing lactate, glucose and ethanol developed for clinical diagnostics [68,69,70,71] can be adapted to quality control tasks in biotechnology processes. Commercial models of a polarimeter, a gas chromatograph, a liquid chromatograph and a capillary electrophoresis system can be an alternative to laboratory biosensors. A polarimeter can be used to measure starch and glucose levels, but it cannot be used for lactate or ethanol analysis. Gas chromatography is selective for ethanol, but lactate, glucose and starch must be analyzed using other methods. Liquid chromatography and capillary electrophoretic systems can analyze glucose, lactate and starch, but ethanol cannot be determined and starch analysis requires hydrolysis prior to analysis. Chromatography and capillary electroporesis systems are expensive.

### 2.6. Biosensor Application

The accuracy of the analysis results obtained using developed receptor elements based on enzymes immobilized in a composite of carbon nanotubes and BSA modified with neutral red was assessed using the “introduced–found” method with state standard samples of lactate, glucose, ethanol and starch. The concentrations of the specified analytes found with the developed biosensor were compared with those declared by the manufacturer. Statistical processing of the results (Student’s *t*-test) showed that there was no significant difference between the mean values of the found values and the declared values. For testing the biosensors, we selected the following products: wine, kvass and fermentation mass. For each series of samples, we determined the content of glucose, lactate or ethanol using a biosensor and a reference method. The results are presented in Table S2. As a result of the statistical analysis of measurement results using the modified Student’s *t*-test, it was found that the concentrations of substances to be measured obtained using developed printed electrodes and standard methods differed insignificantly. The use of printed electrodes in combination with proposed modifications and immobilization techniques makes it possible to produce biosensors with excellent analytical and metrological performance which can be utilized to determine the levels of lactate, glucose, ethanol and starch in fermentation mediums.

## 3. Conclusions

In this investigation we developed a series of hybrid nanocomposite materials comprising redox-active gels anchored in BSA gels with phenazine particles and CNT for the purpose of constructing biosensor. The electrochemical potential of the composite based on neutral red-modified BSA and carbon nanotubes for the immobilization of glucose oxidase, alkyl oxidase, lactate oxidase and joint immobilization of γ-amylase and glucose oxidase was shown. The efficiency of bioelectrocatalysis was confirmed by cyclic voltaperometry: the rate constants of the interaction of covalently bound neutral red with enzymes were 17,000 ± 2000; 2300 ± 700; 1820 ± 60; 4000 ± 200 dm^3^/mol·s for glucose oxidase, alkyl oxidase, lactate oxidase and a combination of γ-amylase and glucose oxidase enzymes, respectively. The impedance spectroscopy method showed that the charge transfer resistance Rct decreased when the substrate was added to the cell, and the amperometry method showed that at a given potential, an analytical signal was generated in the system, the value of which depended on the concentration of the introduced analyte. For each developed receptor element, the working temperature of the analysis was found: 50 °C for monitoring glucose, 35 °C for ethanol, 35–40 °C for lactate, 40–60 °C for starch; the optimal pH of the working electrolyte for using the receptor elements for quality control of biotechnological processes is 7, and the concentration of the sodium-potassium phosphate buffer is 0.033 M. The developed receptor elements are not inferior to analogues: they allow the determination of the contents of starch, glucose, lactate and ethanol in their combined presence in fermentation mass, wine, kvass the analysis time is less than 3 min, and using a redox-active polymer as a matrix increases the long-term stability of the analytical system by preventing leaching of electroactive particles. The limitation of the system is that it requires manual reconnection of electrodes in order to analyze each component. It would be necessary to develop a potentiostat that can simultaneously control four electrodes and create software for data processing.

## 4. Materials and Methods

### 4.1. Reagents and Materials

In the work, graphite printed electrodes were used, which contain a working electrode (diameter 4 mm), an auxiliary electrode and a reference electrode (ColorElectronics, Moscow, Russia). The polymer was formed using BSA (Helicon, Moscow, Russia), glutaraldehyde (25%, Scharlab, Barcelona, Spain), as well as phenazine derivatives (F) as electroactive particles: Nile blue (NB), neutral red (NR), thionin (TH), cresyl blue (CB), azure A (AA), safranin (SN), phenosafranine (PHN), toluidine blue (TB) (Dia-M, Moscow, Russia). A water dispersion (2.5%) single-wall carbon nanotube (LTD «CARBON HGG», Chernogolovka, Russia) was used for electrode modification. D-glucose, starch, lactic acid, ethanol were taken as substrates (Dia-M, Moscow, Russia). Salts KH_2_PO_4_ and Na_2_HPO_4_ (Dia-M, Moscow, Russia) were used to prepare the buffer solution. The following enzymes were used to modify the electrodes: glucose oxidase from *Aspergillus niger* (Sigma-Aldrich, St. Louis, MO, USA, activity 150 U/mg), alcohol oxidase (Aladdin Scientific, Shanghai, China, activity 10 U/mg), lactate oxidase (Macklin, Shanghai, China, activity 139 U/mg), amyloglucosidase (Sigma-Aldrich, St. Louis, MO, USA, activity 60 U/mg).

### 4.2. Formation of Working Electrodes

#### 4.2.1. Formation of a BSA-Modified with Phenazine Electrode (BSA-F Electrode: BSA-NB, BSA-NR, BSA-TH, BSA-CB, BSA-AA, BSA-SN, BSA-PHN, BSA-TB)

To a solution comprising 0.0035 g of BSA, 50 μL of phosphate buffer with a pH of 6.8, and 5 μL of a saturated phenazine solution, were added. The mixture was then agitated for two minutes. Subsequently, 7.5 μL of glutaraldehyde (Scharlab S.L., Barcelona, Spain, soulution 25%) was supplied to the solution, and the mixture was agitated for 30 s. The resulting solution was then applied to the working surface of the electrode in a volume of 3 μL and allowed to dry completely.

#### 4.2.2. Formation of Electrode Containing CNT and BSA-Modified with Phenazine Gel (CNT-BSA-NB, CNT-BSA-NR)

To produce CNT, a sample of the initial nanotube suspension weighing 0.0050 g was dissolved in 50 μL of deionized water, using ultrasonic agitation for a period of two hours. Subsequently, 50 μL of phosphate buffer with a pH of 6.8, 10 μL of the nanotube suspension, 5 μL of a saturated phenazine solution were added to 0.0035 g of BSA. The mixture was stirred for two minutes. After that, 7.5 μL of a 25% glutaraldehyde solution was supplied to the mixture, and it was stirred for 30 s. The resulting solution was then applied to the electrode in an amount of 3 μL and allowed to dry completely.

#### 4.2.3. Formation of Enzyme Electrodes (GOx-CNT-BSA-NR, LOx-CNT-BSA-NR, AOx-CNT-BSA-NR, (AOx + Am)-CNT-BSA-NR)

Enzyme preparations were prepared by dissolving 0.0025 g of the enzyme in 100 μL of sodium-potassium phosphate buffer solution (pH 6.0) with vigorous stirring for 2 min. Then, 50 μL of phosphate buffer (pH 6.8), 10 μL of nanotube suspension, 5 μL of saturated NR solution and 20 μL of enzyme preparation solution were supplied to 0.0035 g of BSA. The resulting mixture was shaken for 2 min. Then 7.5 μL of 25% glutaraldehyde was added to the solution and shaken for 30 s. The resulting mixture was applied to the electrode in an amount of 3 μL and left until completely dry (electrodes GOx-CNT-BSA-NR, LOx-CNT-BSA-NR, AOx-CNT-BSA-NR). The (AOx + Am)-CNT-BSA-NR electrode was prepared by adding 10 μL of GOx and Am to the composition.

### 4.3. Electrochemical Measurements (CV and EIS)

Cyclic voltamperograms were captured using a Corrtest galvanopotentiostat (Wuhan Cortest Instruments Corp., Wuhan, China) configured with a three-electrode setup. Cyclic voltammograms were collected at scan rates ranging from 10 to 250 mV/s in a phosphate buffer at a pH of 6.8.

Impedance spectroscopy measurements were conducted using the same Corrtest galvanopotentiostat and three-electrode cell system, utilizing a potassium-sodium phosphate buffer (pH = 6.8) and applying a specific potential.

### 4.4. 3D-Structure Analysis by Scanning Electron Microscopy

The phenazine-modified gels were examined by a scanning electron microscopy (SEM). Samples were secured to an aluminum platform with double-sided carbon tape. Using a C156RS magnetron sputtering device (TechnoInfo, Moscow, Russia) in an argon atmosphere, 10 nm of chromium was deposited on the samples. The surface of the modified polymer was examined using a Hitachi TM 4000 Plus scanning electron microscope (Hitachi High-Tech Corporation, Tokyo, Japan). Images were captured in secondary electron mode with an accelerating voltage set to 15 kV. SEM investigations, coupled with an energy-dispersive X-ray spectroscopy (SEM-EDS), were performed using an EDX attachment (Bruker, Karlsruhe, Germany) at an accelerating voltage of 20 kV.

### 4.5. Chemical Structured by IR and Raman Spectroscopy

The infrared (IR) spectra of the modified gels were acquired using a Fourier infrared spectrometer (InfaLUM FT-08; Lumex, Saint Petersburg, Russia). The analysis process began with the preparation of a powdered sample of the gel. A mixture of KBr and gel was finely ground in an agate mortar and subsequently pressed into a disk using a laboratory hydraulic press PGR-10 (LabTools, Saint Petersburg, Russia). This disk was then placed into the Fourier spectrometer to measure the absorption spectrum of the resultant substance. Raman spectra were obtained using an M532 Raman microscope (Spektr-M, Chernogolovka, Russia) equipped with a He–Ne laser, which emitted excitation light at a wavelength of 532 nm.

### 4.6. Biosensor Multichannel Measurements

Signal recordings were conducted with an EmStat potentiostat (PalmSens, Houten, The Netherlands). The working potential set for the enzyme electrodes was −0.40 V. The measurements were performed in a 33 mM sodium potassium phosphate buffer at pH 6.8 within a 4 mL cuvette, with magnetic stirring (Ecros, Saint Petersburg, Russia) at 200 rpm. For each specific substance, an electrode was modified with a different enzyme gel composition. This meant that a biosensor contained four different electrodes, each electrode was calibrated to a specific standard (lactic acid, glucose, ethanol or starch), and the signals from each electrode were processed separately. The analytical signal (biosensor response) was defined as the change in current passing through the working electrode, measured from the moment the substrate was added to the point of reaching the highest current level. Following each measurement, the electrode was rinsed with buffer solution for 1 to 2 min.

### 4.7. Determination of Lactic Acid, Glucose and Starch Content by Capillary Electrophoresis

Measurements were conducted using a Kapel-104T capillary electrophoresis system (Lumex, Saint Petersburg, Russia) operating under negative high-voltage polarity. The system was fitted with a quartz capillary (inner diameter of 50 μm, effective length of 65 cm, and total length of 75 cm) and a photometer set to measure at 254 nm. Prior to analysis, the samples underwent filtration through a membrane Millipore (Sigma-Aldrich, St. Louis, MO, USA). The analytical signal was determined based on the peak area. To assess the starch content, a preliminary hydrolysis of the sample was performed. This involved heating the sample in a water bath with 1.2% hydrochloric acid for 15 min, after which protein substances were precipitated, the mixture was filtered and the analysis continued as previously outlined.

### 4.8. Determination of Ethyl Alcohol Content by Gas Chromatography

The concentration of ethyl alcohol was measured using gas–liquid chromatography on a Crystal-5000.2 chromatograph (ZAO SKB Khromatek, Podolsk, Russia) equipped with a flame ionization detector and a DB-FFAP capillary column (50 m × 0.32 mm × 0.50 μm) (Agilent, Santa Clara, CA, USA). The parameters for the analysis were set as follows: the column thermostat temperature was −70 °C, the evaporator temperature was 200 °C, the detector temperature was 250 °C and the flow gas rate of helium was 0.10 dm^3^/h.

## Figures and Tables

**Table 1:** Electrochemical parameters of redox-active gels.

Redox Gel	Results of Cyclic Voltammetry			Impedance			
	E, B	k_s_, s^−1^	k_int_, L/mol⋅s	Rs, Ohm	Cdl, μF	α_dl_	Rct, MOhm
BSA-NB	0.5	0.024 ± 0.006	6300 ± 1200	295	0.08	0.92	10
BSA-NR	−0.7	0.0012 ± 0.0001	5840 ± 60	264	0.09	0.91	3.6
BSA-TH	−0.3	0.0090 ± 0.0006	2000 ± 100	298	0.07	0.92	12
BSA-CB	−0.5	0.018 ± 0.004	2300 ± 100	257	0.10	0.89	6.6
BSA-TB	−0.6	0.00077 ± 0.00004	-	261	0.15	0.89	1.8
BSA-PHN	−0.5	0.00010 ± 0.00005	2000 ± 100	311	0.12	0.89	1.5
BSA-SN	−0.6	0.0116 ± 0.0006	4300 ± 300	292	0.07	0.93	5.5
BSA-AA	−0.3	0.045 ± 0.002	129 ± 6	276	0.18	0.94	1.4

**Table 2:** Rate constants of interaction of mediators with the enzyme for printed electrodes.

Parameter	BSA-NB-CNT /BSA-NB	BSA-NR-CNT /BSA-NR	Graphene Oxide Based Nanocomposite [47]	Electrode Based on Gold Nanoparticles [48]
Rate constant of heterogeneous electron transfer, (sm·s^−1^)	0.022 ± 0.001 /0.024 ± 0.006	0.0020 ± 0.0001 /0.0012 ± 0.0001	3.5	1.01
Rate constant of interaction with GOx, L/mol·s	2000 ± 300 /6300 ± 1200	17,000 ± 2000 /2840 ± 60	- *	- *

- * Data not specified.

**Table 3:** Main characteristics of the developed biosensors and analogues.

	Receptor System	Advantages	Analyte	Main Characteristics	Ref.
1.	GOx/BSA-NR-CNT	Multichannel, portable, rapid, sensitive, long-term	Glucose	R: 0.035–0.60 mM; O: 2.7% T: 1–2 min; L: 30 days	This work
	AOx/BSA-NR-CNT		Ethanol	R: 2.3–9 mM; O: 2.8% T: 1–2 min; L: 7 days	
	LOx/BSA-NR-CNT		Lactic acid	R: 15–57 mM; O: 7.6% T: 1–2 min; L: 7 days	
	Am + GOx/BSA-NR-CNT		Starch	R: 2–29 mg/L; O: 4.5% T: 1–2 min; L: 14 days.	
2.	GOx/BSA-NR	Rapid, sensitive, portable	Glucose	R: 0.6–2.7 mM; O: 1.6 %; T: 1–3 min; L: 12 days	[66]
3.	GOx/BSA-ferrocene-CNT	Rapid, sensitive, portable	Glucose	R: 0.1–1.8 mM; O: 5.08 %; T: 1–3 min; L: 16 days	[66]
4.	GOx/Ferrocene, BSA	Two channel, portable, rapid, sensitive	Glucose	R: 0–1.5 mM; L: 1 day	[67]
	LOx/Ferrocene, BSA		Lactate	R: 0–2 mM; L: 1 day	
5.	GOx/reduced graphene oxide	Two channel, rapid, portable	Glucose	R: 2.0–100 mg/L (0.01–0.55 mM)	[26]
	GOx + Am/reduced graphene oxide		Starch	R: 50–3500 mg/L	
6.	AOx/2,3,5,6-tetrachloro-1,4-benzoquinone, lipid layer, multi-walled carbon nanotubes	Rapid, sensitive, portable	Ethanol	R: 0.2–13 mM	[68]
7.	LOx/Chitosan, gold nanoparticles	Rapid, sensitive, portable	Lactate	R: 0–30 mM	[69]
8.	AOx/Chitosan	Two channel, portable, rapid, sensitive	Ethanol	R: 0–40 mM	[70]
	GOx/Chitosan		Glucose	R: 0–0.16 mM	
9.	GOx/platinum nanoparticles	Two channel, portable, rapid, sensitive, long-term	Glucose	R: 0.025–0.300 mM; O: 4.2% L: 30 days	[71]
	LOx/platinum nanoparticles		Lactate	R: 5–35 mM; O: 2.8% L: 30 days	

R—range of detectable concentrations; O—operational stability; T—single measurement time; L—long-term stability.

## Data Availability

The data that support the findings of this study are available on request from the corresponding author.

## Supplementary Materials

The following supporting information can be downloaded at https://www.mdpi.com/article/10.3390/gels11050355/s1, Figure S1: IR spectra; Figure S2: Raman spectra; Figure S3: SEM and EDX of the redox-active polymer: A. BSA-AA, B. BSA-CB, C. BSA-NR, D. BSA-NB, E. BSA-TH, F. BSA-TB, G. BSA-PHN; Figure S4: Electrochemical properties of polymers CV and heterogeneous constant rate calculation for: A. BSA-NB, B. BSA-NR, C. BSA-TH, D. BSA-CB, E. BSA-PHN, F. BSA-AA, G. BSA-SN, H. BSA-TB, I. BSA (Control), J. EIS of BSA (Control); Text S1: Heterogeneous electron transfer constant rate determination; Text S2: constant rate determination; Figure S5: Dependence (Id/Ik) on 1/ν^1/2^ for electrodes based on: A. BSA-NC; B. BSA-NR; C. BSA-TH; D. BSA-CB, E. BSA-SN, F. BSA-PHN,G. BSA-AA.; Figure S6: SEM and EDX of the BSA-NB-CNT composite; Figure S7: CV of composite for heterogeneous constant rate calculation A. BSA-NB-CNT; B. BSA-NR-CNT; Figure S8. CV of the BSA-NB-CNT composite for constant rate calculation; Figure S9. Dependence (Id/Ik) on 1/ν^1/2^ for electrodes based on: A. BSA-NB-CNT; B. BSA-NR-CNT; Figure S10. Electrochemical impedance spectra of BSA-NB-CNT composite and equivalent electrical circuit used for fitting the spectra. Table S1. Rate constants of interaction of mediators with enzymes. Figure S11. Effect of temperature on the response of a biosensor for the determination of A. Ethanol; B. Lactate; C. Starch. Figure S12. Effect of pH on the response of the biosensor for the determination of A. Ethanol; B. Lactate; C. Starch. Figure S13. Effect of NaCl concentration on the response of the biosensor for the determination of A. Ethanol; B. Lactate; C. Starch. Figure S14. Effect of phosphate buffer solution salt concentration on the response of the biosensor for the determination of A. Ethanol; B. Lactate; C. Starch. Figure S15. Effect of heavy metal ions on the response of the biosensor for the determination of A. Ethanol; B. Lactate; C. Starch.

## References

[1] Qin S., Sun X., Zhao X. (2025). Advances in smartphone-based biosensors for food testing. Curr. Opin. Food Sci..

[2] Hosseinikebria S., Khazaei M., Dervisevic M., Judicpa M.A., Tian J., Razal J.M., Voelcker N.H., Nilghaz A. (2025). Electrochemical biosensors: The beacon for food safety and quality. Food Chem..

[3] Inês A., Cosme F. (2025). Biosensors for Detecting Food Contaminants—An Overview. Processes.

[4] Nath S. (2024). Advancements in food quality monitoring: Integrating biosensors for precision detection. Sustain. Food Technol..

[5] Meliana C., Liu J., Show P.L., Low S.S. (2024). Biosensor in smart food traceability system for food safety and security. Bioengineered.

[6] Wijayanti S.D., Tsvik L., Haltrich D. (2023). Recent Advances in Electrochemical Enzyme-Based Biosensors for Food and Beverage Analysis. Foods.

[7] Sivashankar S., Kumar N.S., Kiran R., Mazumder N. (2020). Amperometric Biosensors as an Analytical Tool in Food, Dairy and Fermentation Industries. Int. J. Pharm. Sci. Rev. Res..

[8] Malik S., Singh J., Saini K., Chaudhary V., Umar A., Ibrahim A.A., Akbar S., Baskoutas S. (2024). Paper-based sensors: Affordable, versatile, and emerging analyte detection platforms. Anal. Methods.

[9] Ansari A.A., Malhotra B.D. (2022). Current progress in organic–inorganic hetero-nano-interfaces based electrochemical biosensors for healthcare monitoring. Coord. Chem. Rev..

[10] Guesmi S., Moulaee K., Bressi V., Kahri H., Khaskhoussi A., Espro C., Barhoumi H., Neri G. (2024). Non-enzymatic amperometric glucose sensing by novel Cu-MOF synthesized at room temperature. Mater. Adv..

[11] Dong Q., Ling C., Zhao S., Tang X., Zhang Y., Xing Y., Yu H., Huang K., Zou Z., Xiong X. (2023). One-step rapid synthesis of Ni_0.5_Co_0.5_-CPO-27 nanorod array with oxygen vacancies based on DBD microplasma: As an effective non-enzymatic glucose sensor for beverage and human serum. Food Chem..

[12] Josypcuk B., Tvorynska S. (2024). Electrochemical flow-through biosensors based on microfiber enzymatic filter discs placed at printed electrodes. Bioelectrochemistry.

[13] Kuznetsova L.S., Arlyapov V.A., Kamanina O.A., Lantsova E.A., Tarasov S.E., Reshetilov A.N. (2022). Development of Nanocomposite Materials Based on Conductive Polymers for Using in Glucose Biosensor. Polymers.

[14] Kuznetsova L.S., Ivanova K.D., Lantsova E.A., Saverina E.A., Kharkova A.S., Guetnga C., Lipkin M.S., Kamanina O.A., Kozlova T.V., Popova N.M. (2024). Cross-Disciplinary Glucose Biosensors: An ORMOSIL/Enzyme Material for Enhanced Detection. ACS Appl. Polym. Mater..

[15] Kamanina O.A., Kamanin S.S., Kharkova A.S., Arlyapov V.A. (2019). Glucose biosensor based on screen-printed electrode modified with silicone sol–gel conducting matrix containing carbon nanotubes. 3 Biotech.

[16] Koley P., Jakku R., Hosseinnejad T., Periasamy S., Bhargava S.K. (2024). Immobilizing nanozymes on 3D-printed metal substrates for enhanced peroxidase-like activity and trace-level glucose detection. Nanoscale.

[17] Stasyuk N., Demkiv O., Gayda G., Zakalska O., Zakalskiy A., Serkiz R., Kavetskyy T., Gonchar M. (2022). Reusable alcohol oxidase–nPtCu/alginate beads for highly sensitive ethanol assay in beverages. RSC Adv..

[18] Istrate O.-M., Rotariu L., Bala C. (2021). Amperometric L-Lactate Biosensor Based upon a Gold Nanoparticles/Reduced Graphene Oxide/Polyallylamine Hydrochloride Modified Screen-Printed Graphite Electrode. Chemosensors.

[19] Wei Q., Dong Q., Pu H. (2023). Multiplex Surface-Enhanced Raman Scattering: An Emerging Tool for Multicomponent Detection of Food Contaminants. Biosensors.

[20] Smutok O., Kavetskyy T., Prokopiv T., Serkiz R., Wojnarowska-Nowak R., Šauša O., Novák I., Berek D., Melman A., Gonchar M. (2021). New micro/nanocomposite with peroxidase-like activity in construction of oxidases-based amperometric biosensors for ethanol and glucose analysis. Anal. Chim. Acta.

[21] (2012). Safety Requirements for Food Additives, Flavorings and Technological Aids. TRCU (Technical Regulations of the Customs Union).

[22] Röhlen D.L., Pilas J., Dahmen M., Keusgen M., Selmer T., Schöning M.J. (2018). Toward a Hybrid Biosensor System for Analysis of Organic and Volatile Fatty Acids in Fermentation Processes. Front Chem..

[23] Rodriguez-Mendez M.L. (2023). Nanostructured thin films as electrochemical sensors and biosensors for milk analysis. Sens. Actuators Rep..

[24] Ozoglu O., Uzunoglu A., Unal M.A., Gumustas M., Ozkan S.A., Korukluoglu M., Altuntas E.G. (2023). Electrochemical Detection of Lactate Produced by Foodborne Presumptive Lactic Acid Bacteria. J. Biosci. Bioeng..

[25] Tsvik L., Zhang S., O’hare D., Haltrich D., Sützl L. (2024). More Than One Enzyme: Exploring Alternative FMN-Dependent L-Lactate Oxidases for Biosensor Development. ACS Omega.

[26] Liu J., Lang Q., Liang B., Zheng Z., Zhang Y., Liu A. (2022). Sensitive electrochemical sequential enzyme biosensor for glucose and starch based on glucoamylase- and glucose oxidase-controllably co-displayed yeast recombinant. Anal. Chim. Acta.

[27] Ma J., Fei Y., Zhang J., Wu H. (2025). Wearable multiple sensing platform for enhanced biomolecules monitoring in food. Food Chem..

[28] Chen X., Chen Y., Tang D., Li M., Lu Y., Cao Y., Zhao Q., Jiang S., Liu W., Jiang L. (2025). Recent advances in bioinspired multienzyme engineering for food applications. Trends Food Sci. Technol..

[29] Rogala A., Nestorowicz R., Jerzyk E. (2024). Internet of Things in the Food Industry: Challenges and Opportunities for the Internet of Food Things.

[30] Kamanin S.S., Arlyapov V.A., Machulin A.V., Alferov V.A., Reshetilov A.N. (2015). Biosensors based on modified screen-printed enzyme electrodes for monitoring of fermentation processes. Russ. J. Appl. Chem..

[31] Arlyapov V.A., Kamanina O.A., Kamanin S.S., Reshetilov A.N., Shvets V.I. (2019). Monitoring of Biotechnological Processes by Enzyme Electrodes Modified with Carbon Nanotubes. Appl. Biochem. Microbiol..

[32] Damala P., Tiuftiakov N.Y., Bakker E. (2024). Avoiding Potential Pitfalls in Designing Wired Glucose Biosensors. ACS Sens..

[33] Weng Y., Chen R., Hui Y., Chen D., Zhao C.-X. (2024). Boosting Enzyme Activity in Enzyme Metal–Organic Framework Composites. Chem Bio Eng..

[34] Dalkiran B., Brett C.M.A. (2021). Polyphenazine and polytriphenylmethane redox polymer/nanomaterial–based electrochemical sensors and biosensors: A review. Microchim. Acta.

[35] Kuznetsova L.S., Arlyapov V.A., Plekhanova Y.V., Tarasov S.E., Kharkova A.S., Saverina E.A., Reshetilov A.N. (2023). Conductive Polymers and Their Nanocomposites: Application Features in Biosensors and Biofuel Cells. Polymers.

[36] Pisarevskaya E.Y., Ozkan S.Z., Petrov V.A., Klyuev A.L., Efimov O.N., Karpacheva G.P. (2024). Electrochemical properties of novel redox active electrode coatings based on heterocyclic polyazines and its nanocomposites with carbon nanomaterials. J. Electroanal. Chem..

[37] Kulikova T., Shiabiev I., Padnya P., Rogov A., Evtugyn G., Stoikov I., Porfireva A. (2023). Impedimetric DNA Sensor Based on Electropolymerized N-Phenylaminophenothiazine and Thiacalix [4] arene Tetraacids for Doxorubicin Determination. Biosensors.

[38] Kharkova A.S., Medvedeva A.S., Kuznetsova L.S., Gertsen M.M., Kolesov V.V., Arlyapov V.A., Reshetilov A.N. (2024). A “2-in-1” Bioanalytical System Based on Nanocomposite Conductive Polymers for Early Detection of Surface Water Pollution. Polymers.

[39] Lavrova T., Kharkova A., Perchikov R., Gertsen M., Shadrin A., Arlyapov V. (2025). A Highly Sensitive Test System for Measuring the Phenolic Index of Wastewater Based on a Biocompatible Composite Material with Carbon Nanotubes. J. Polym. Environ..

[40] Arlyapov V.A., Khar’kova A.S., Abramova T.N., Kuznetsova L.S., Ilyukhina A.S., Zaitsev M.G., Machulin A.V., Reshetilov A.N. (2020). A Hybrid Redox-Active Polymer Based on Bovine Serum Albumin, Ferrocene, Carboxylated Carbon Nanotubes, and Glucose Oxidase. J. Anal. Chem..

[41] Perchikov R.N., Provotorova D.V., Kharkova A.S., Arlyapov V.A., Medvedeva A.S., Machulin A.V., Filonov A.E., Reshetilov A.N. (2022). Bioanalytical System for Determining the Phenol Index Based on *Pseudomonas putida* BS394(pBS216) Bacteria Immobilized in a Redox-Active Biocompatible Composite Polymer “Bovine Serum Albumin–Ferrocene–Carbon Nanotubes”. Polymers.

[42] Sergeevna K.A., Vladimirovna P.D., Valerievich M.A., Alekseevich A.V. (2023). Acceptor properties of “carbon nanotubes–redox-active polymer based on bovine serum albumin modified with ferrocenecarboxaldehyde” composite for creating a BOD biosensor with *Blastobotrys adeninivorans* BKM Y-2677 yeast. 3 Biotech.

[43] Sergeev A.V., Rudyak V.Y., Samodelkin R.A., Kozhunova E.Y., Chertovich A.V. (2025). Optimizing the charge transport in redox-active gels: A computational study. Soft Matter.

[44] Yang X., He Q., Guo F., Sun X., Zhang J., Chen Y. (2021). Impacts of carbon-based nanomaterials on nutrient removal in constructed wetlands: Microbial community structure, enzyme activities, and metabolism process. J. Hazard. Mater..

[45] Wang X., Wang Q. (2021). Enzyme-laden bioactive hydrogel for biocatalytic monitoring and regulation. Acc. Chem. Res..

[46] Shen J., Zhang S., Fang X., Salmon S. (2022). Advances in 3D gel printing for enzyme immobilization. Gels.

[47] Wang B., Wang X., He Z., Zhao X., Wang L. (2019). Direct Electrochemistry of Glucose Oxidase on a GrapheneGraphene Oxide Nanocomposite-Modified Electrode for a Glucose Biosensor. Int. J. Electrochem. Sci..

[48] Niu Y., Liu J., Chen W., Yin C., Weng W., Li X., Wang X., Li G., Sun W. (2018). A direct electron transfer biosensor based on a horseradish peroxidase and gold nanotriangle modified electrode and electrocatalysis. Anal. Methods.

[49] Jadàn-Piedra F., Cevallos-Mendoza J.E., Delgado J.M.V., Mendoza V.S., Mero M.L., Zambrano R.A.R., Utreras J.A.D., Rodríguez-Gamez M., Jadàn-Piedra C. (2023). Selective determination of methanol and ethanol using a sensor based on alcohol oxidase immobilized on a cassava biopolymer. Biosens. Bioelectron. X.

[50] Del Moral S., Barradas-Dermitz D.M., Aguilar-Uscanga M.G. (2018). Production and biochemical characterization of α-glucosidase from *Aspergillus niger* ITV-01 isolated from sugar cane bagasse. 3 Biotech.

[51] Arslan H., Özdemir M., Zengin H., Zengin G. (2012). Glucose Biosensing at Carbon Paste Electrodes Containing Polyaniline-Silicon dioxide Composite. Int. J. Electrochem. Sci..

[52] Prasanna Kumar S., Parashuram L., Suhas D.P., Krishnaiah P. (2020). Carboxylated graphene-alcohol oxidase thin films modified graphite electrode as an electrochemical sensor for electro-catalytic detection of ethanol. Mater. Sci. Energy Technol..

[53] Klepach H.M., Zakalskiy A.E., Zakalska O.M., Gayda G.Z., Smutok O.V., Gonchar M.V., Barile M. (2021). Alcohol Oxidase from the Methylotrophic Yeast *Ogataea polymorpha*: Isolation, Purification, and Bioanalytical Application. Flavins and Flavoproteins.

[54] Dias C., Fernandes E., Barbosa R.M., Ledo A. (2022). A Platinized Carbon Fiber Microelectrode-Based Oxidase Biosensor for Amperometric Monitoring of Lactate in Brain Slices. Sensors.

[55] Verma P., Singh M., Dhull V. (2023). ZnO/MWCNTs/Au Based Nano Biosensor for Detection of Lactate in Food Samples. Anal. Bioanal. Electrochem..

[56] Donkin R., Robinson S., Sumby K., Harris V., McBryde C., Jiranek V. (2010). Sodium Chloride in Australian Grape Juice and Its Effect on Alcoholic and Malolactic Fermentation. Am. J. Enol. Vitic..

[57] Liu J., Cui Z. (2007). Optimization of operating conditions for glucose oxidation in an enzymatic membrane bioreactor. J. Membr. Sci..

[58] Kagan M., Kivirand K., Rinken T. (2013). Modulation of enzyme catalytic properties and biosensor calibration parameters with chlorides: Studies with glucose oxidase. Enzyme Microb. Technol..

[59] Pohanka M., Zbořil P. (2008). Amperometric Biosensor for D-Lactate Assay. Food Technol. Biotechnol..

[60] Barsan M.M., Brett C.M.A. (2008). An alcohol oxidase biosensor using PNR redox mediator at carbon film electrodes. Talanta.

[61] (1992). Maximum Permissible Concentrations of Heavy Metals and Arsenic in Food Raw Materials and Food Products.

[62] Schiavo D., Neira J.Y., Nóbrega J.A. (2008). Direct determination of Cd, Cu and Pb in wines and grape juices by thermospray flame furnace atomic absorption spectrometry. Talanta.

[63] Redan B.W., Jablonski J.E., Halverson C., Jaganathan J., Mabud A., Jackson L.S. (2019). Factors Affecting Transfer of the Heavy Metals Arsenic, Lead, and Cadmium from Diatomaceous-Earth Filter Aids to Alcoholic Beverages during Laboratory-Scale Filtration. J. Agric. Food Chem..

[64] Scutarașu E.C., Trincă L.C. (2023). Heavy Metals in Foods and Beverages: Global Situation, Health Risks and Reduction Methods. Foods.

[65] Annamalai M., Balu M., Alwarappan S., Lakshmanan R. (2024). A Theoretical Approach to Understand the Nonlinear Processes in Enzymatic Electrochemical Biosensors. J. Phys. Chem. B.

[66] Arlyapov V.A., Kuznetsova L.S., Kharkova A.S., Provotorova D.V., Nenarochkina E.D., Kamanina O.A., Machulin A.V., Ponamoreva O.N., Alferov V.A., Reshetilov A.N. (2022). On the Development of Reagent-free Conductive Nanocomposite Systems for the Modification of Printed Electrodes When Producing Glucose Biosensors. Nanobiotechnol. Rep..

[67] Liu M., Yang M., Wang M., Wang H., Cheng J. (2022). A Flexible Dual-Analyte Electrochemical Biosensor for Salivary Glucose and Lactate Detection. Biosensors.

[68] Wang S., Yao Z., Yang T., Zhang Q., Gao F. (2019). An Enzymatic Electrode Integrated with Alcohol Dehydrogenase and Chloranil in Liquid-Crystalline Cubic Phases on Carbon Nanotubes for Sensitive Amperometric Detection of NADH and Ethanol. J. Electrochem. Soc..

[69] Wang R., Zhai Q., An T., Gong S., Cheng W. (2021). Stretchable gold fiber-based wearable textile electrochemical biosensor for lactate monitoring in sweat. Talanta.

[70] Kim J., Sempionatto J.R., Imani S., Hartel M.C., Barfidokht A., Tang G., Campbell A.S., Mercier P.P., Wang J. (2018). Simultaneous Monitoring of Sweat and Interstitial Fluid Using a Single Wearable Biosensor Platform. Adv. Sci..

[71] He W., Wang C., Wang H., Jian M., Lu W., Liang X., Zhang X., Yang F., Zhang Y. (2019). Integrated textile sensor patch for real-time and multiplex sweat analysis. Sci. Adv..

