# Supplementary material for: Bionanocomposite Four-Channel Biosensor for Rapid and Convenient Monitoring of Glucose, Lactate, Ethanol and Starch
Source: Gels. 2025 May 12;11(5):355. doi: 10.3390/gels11050355 (PMC12111602; doi:10.3390/gels11050355)
Supplement: Supplementary file 1 [file gels-11-00355-s001.zip › gels-3602346-supplementary.pdf]

Bionanocomposite Four-Channel Biosensor for Rapid and Convenient Monitoring of Glucose, Lactate, Ethanol and Starch

Anna Kharkova [1], Lyubov Kuznetsova [1], Roman Perchikov [1], Maria Gertsen [2], Pavel Melnikov [3], Nikolay Zaitsev [4], Jun Zhang [5] and Vyacheslav Arlyapov [1,*]

[1] The Research Center «BioChemTech», Tula State University, 300012 Tula, Russia; anyuta_zaytseva@mail.ru (A.K.); l.s.latunina@gmail.com (L.K.); perchikov_roma@mail.ru (R.P.)
[2] Laboratory of Soil Chemistry and Ecology, Tula State Lev Tolstoy Pedagogical University, 300026 Tula, Russia; mani.gertsen@gmail.com
[3] M.V. Lomonosov Institute of Fine Chemical Technologies, MIREA—Russian Technological University, 119571 Moscow, Russia
[4] Econics-Expert Ltd., Akademika Bakuleva St., 6, 117513 Moscow, Russia
[5] National Key Laboratory of Urban and Rural Water Resources and Water Environment, School of Environment, Harbin Institute of Technology, Harbin 150090, China
* Correspondence: v.a.arlyapov@gmail.com

Table of contents

Figure S1. IR spectra 5

Figure S2. Raman spectra 9

Figure S4. CV of polymers for heterogeneous constant rate calculation: A. BSA-NB, B. BSA-NR, C. BSA-TH, D. BSA-CB, E. BSA-PHN, F. BSA-AA, G. BSA-SN, H. BSA-TB 13

Text S1. Heterogeneous electron transfer constant rate determination 13

Text S2. The rate constant of interaction between the redox gel and enzyme determination 14

Figure S5. Dependence of (I_d/I_k) on $1/v^{1/2}$ for printed electrodes based on: A. BSA-NB;B. BSA-NR; C. BSA-TH; D. BSA-BC; E. BSA-SN; F. BSA-FHS; G. BSA-AA 16

Figure S6. SEM and EDX of the BSA-NB-CNT composite. 16

Figure S7. CV of composite for heterogeneous constant rate calculation A. BSA-NB-CNT composite. B. BSA-NR-CNT 17

Figure S9. Dependence of (I_d/I_k) on $1/v^{1/2}$ for printed electrodes based on: A. BSA-NB-CNT; B. BSA-NR-CNT 17

Figure S10. Electrochemical impedance spectra of *BSA-NB-CNT* composite and equivalent electrical circuit used for fitting the spectra 17

Table S1. The rate constant of interaction between the redox gel and enzymes 17

Figure S11. Effect of temperature on the response of the biosensor for the determination of: A. Ethanol; B. Lactate; C. Starch 18

Figure S12. Effect of pH on the response of the biosensor for the determination of: A. Ethanol; B. Lactate; C. Starch 19

Figure S13. Effect of NaCl concentration on the response of the biosensor for the determination of: A. Ethanol; B. Lactate; C. Starch..19

Figure S14. Effect of concentration of salts of phosphate buffer solution on the response of the biosensor for the determination of: A. Ethanol; B. Lactate; C. Starch....................................19

Figure S15. Effect of heavy metal ions on the response of the biosensor for the determination of: A. Ethanol; B. Lactate; C. Starch..20

Table S2. Results of measuring glucose, lactate, ethanol and starch using the developed biosensor and reference methods..20

Support information

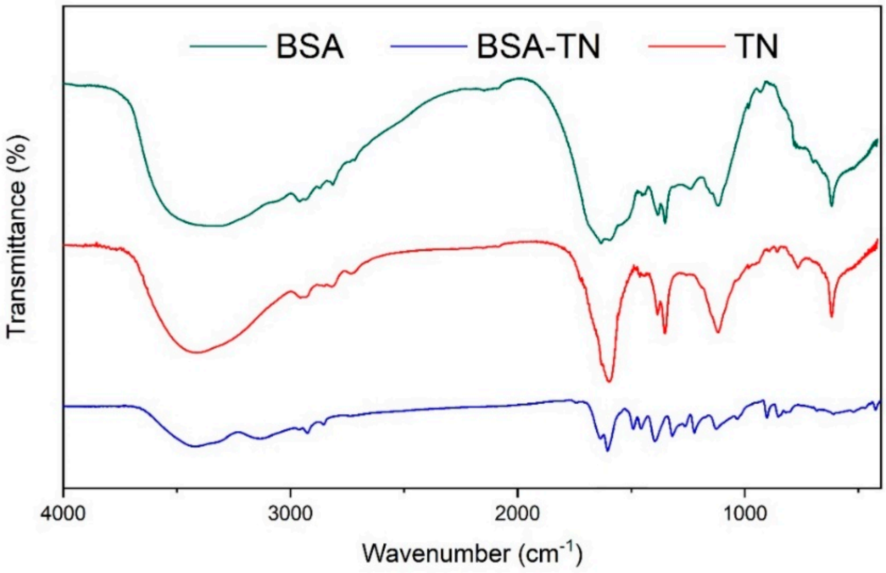

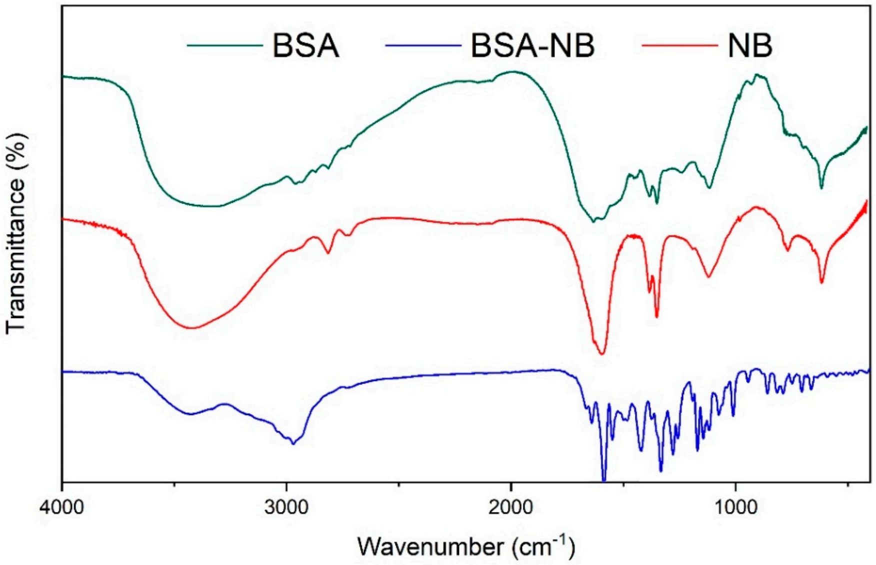

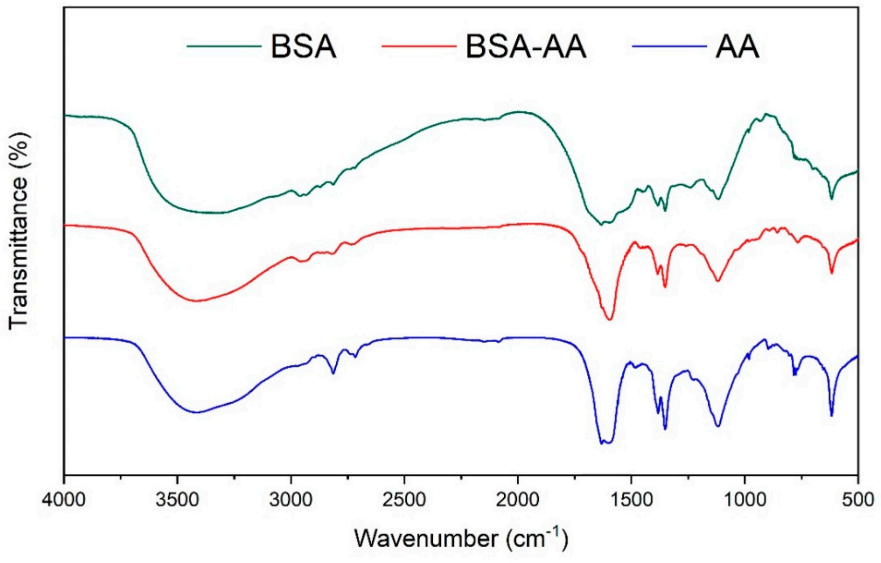

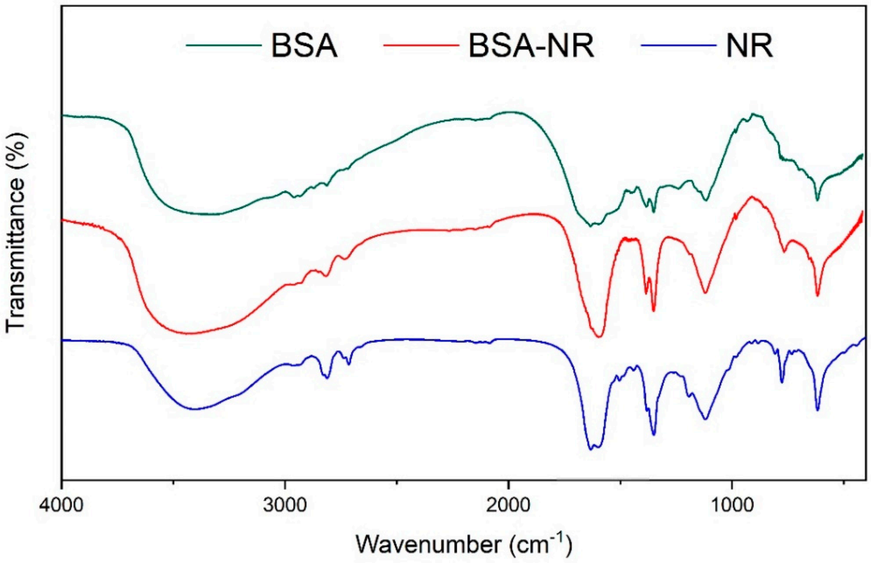
BSA
BSA-NR
NR
Transmittance (%)
4000
3000
2000
1000
Wavenumber (cm-1)

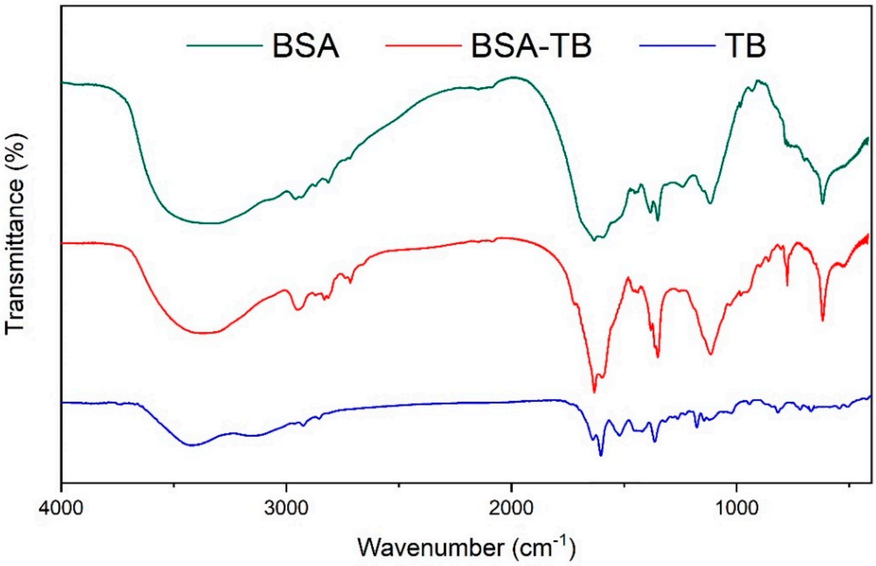
BSA
BSA-TB
TB
Transmittance (%)
4000
3000
2000
1000
Wavenumber (cm-1)

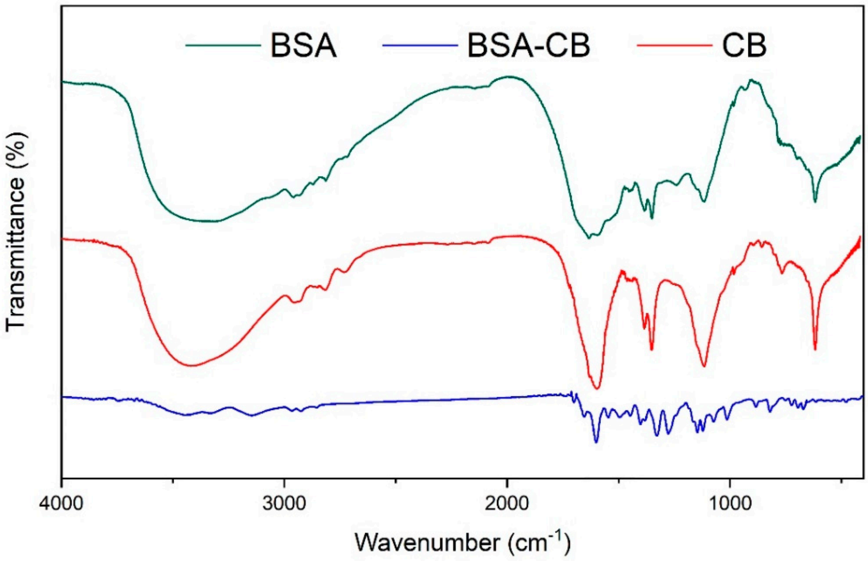
BSA
BSA-CB
CB
Transmittance (%)
4000
3000
2000
1000
Wavenumber (cm-1)

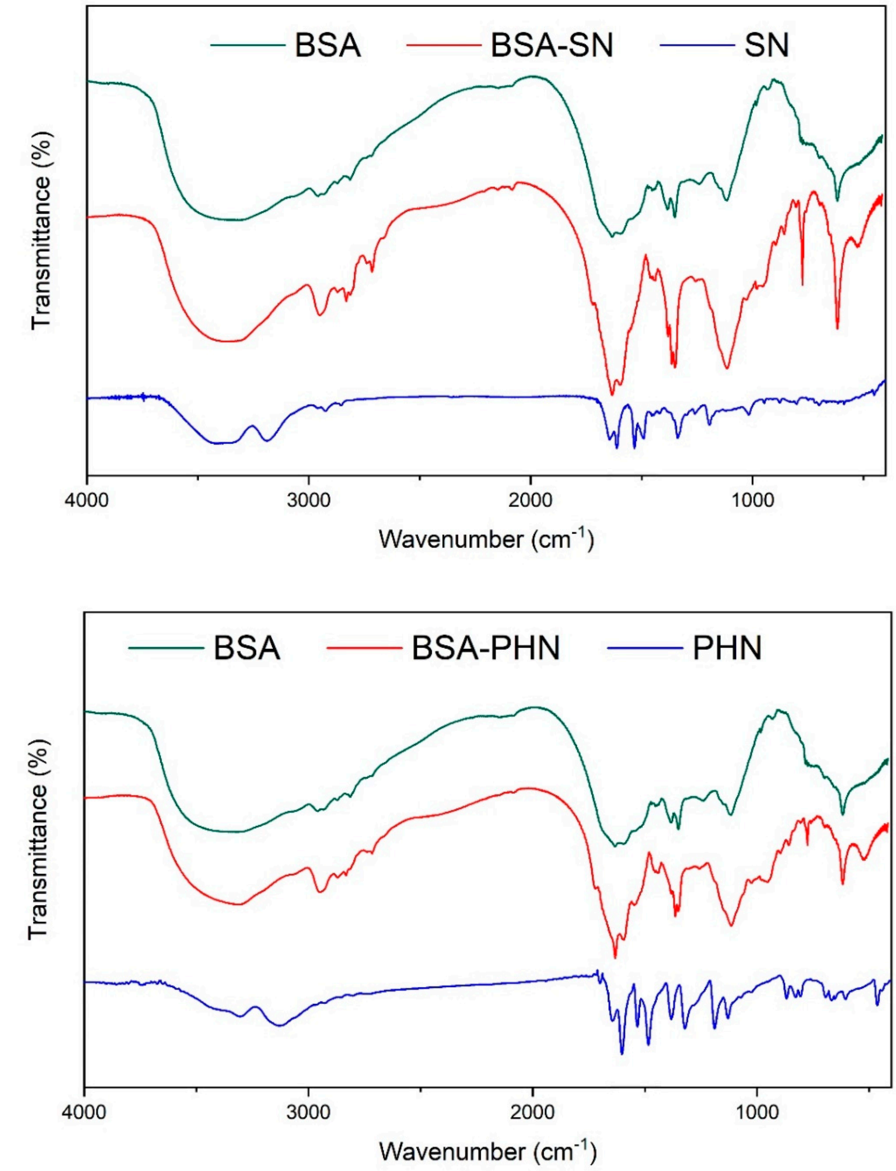

Figure S1. IR spectra

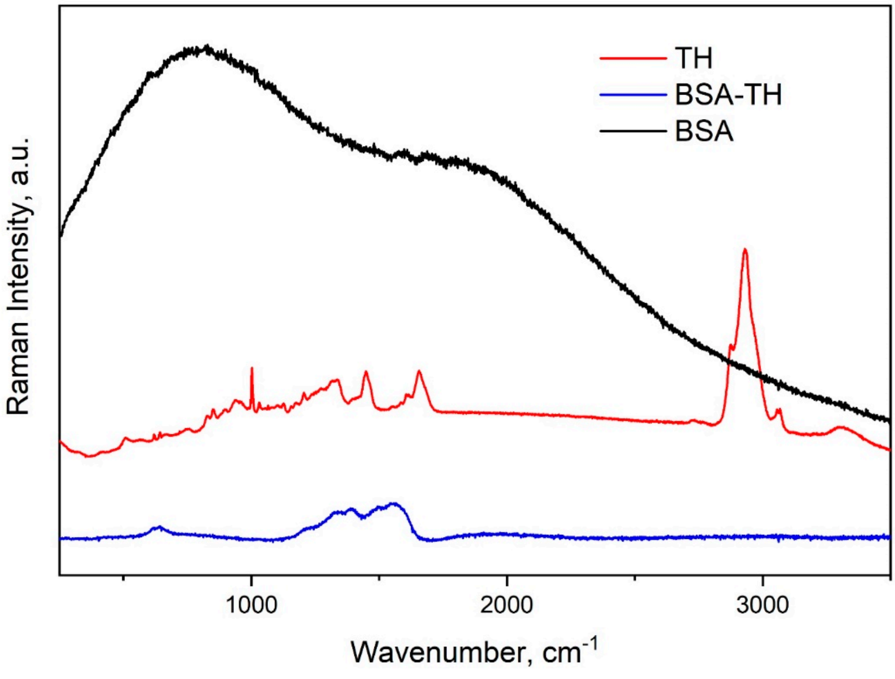

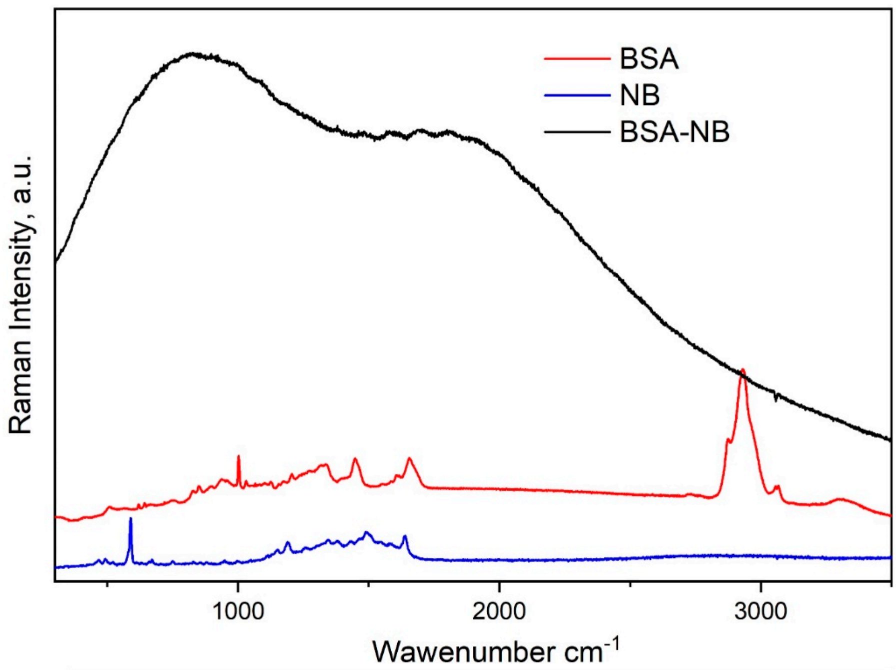
BSA
NB
BSA-NB
Raman Intensity, a.u.
1000
2000
3000
Wawenumber cm-1

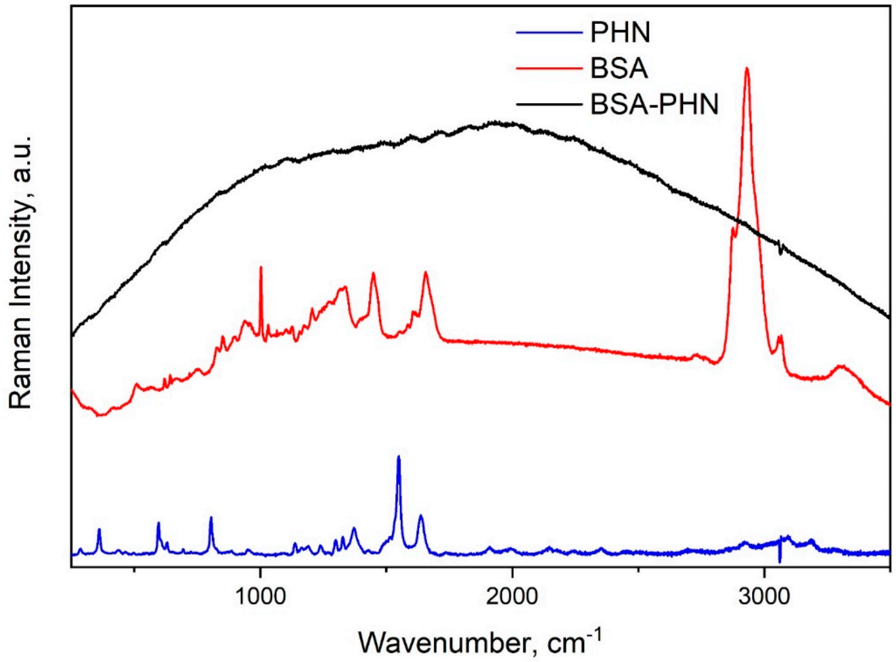
PHN
BSA
BSA-PHN
Raman Intensity, a.u.
1000
2000
3000
Wavenumber, cm-1

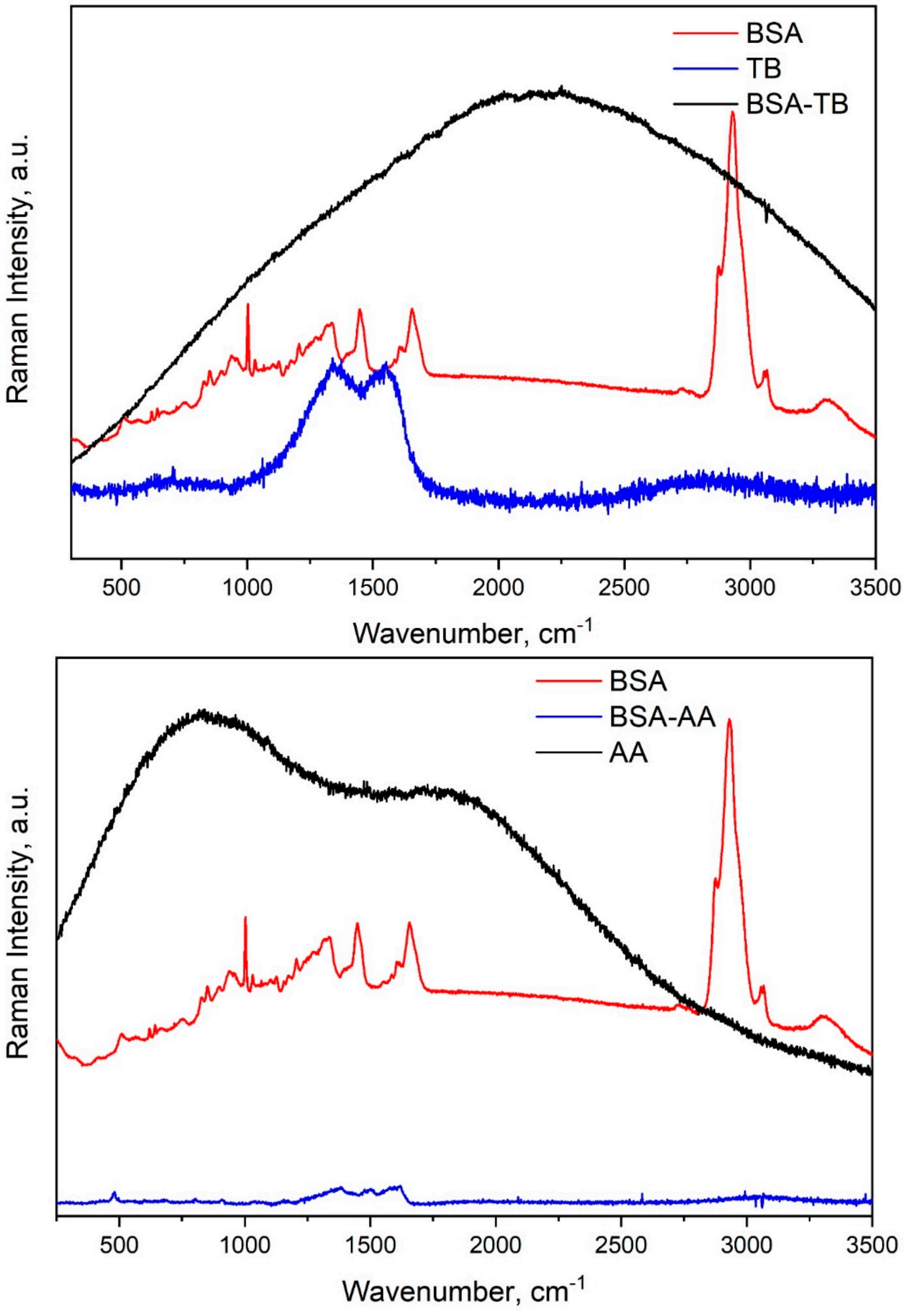
BSA
TB
BSA-TB
Raman Intensity, a.u.
500
1000
1500
2000
2500
3000
3500
Wavenumber, cm-1
BSA
BSA-AA
AA
Raman Intensity, a.u.
500
1000
1500
2000
2500
3000
3500
Wavenumber, cm-1

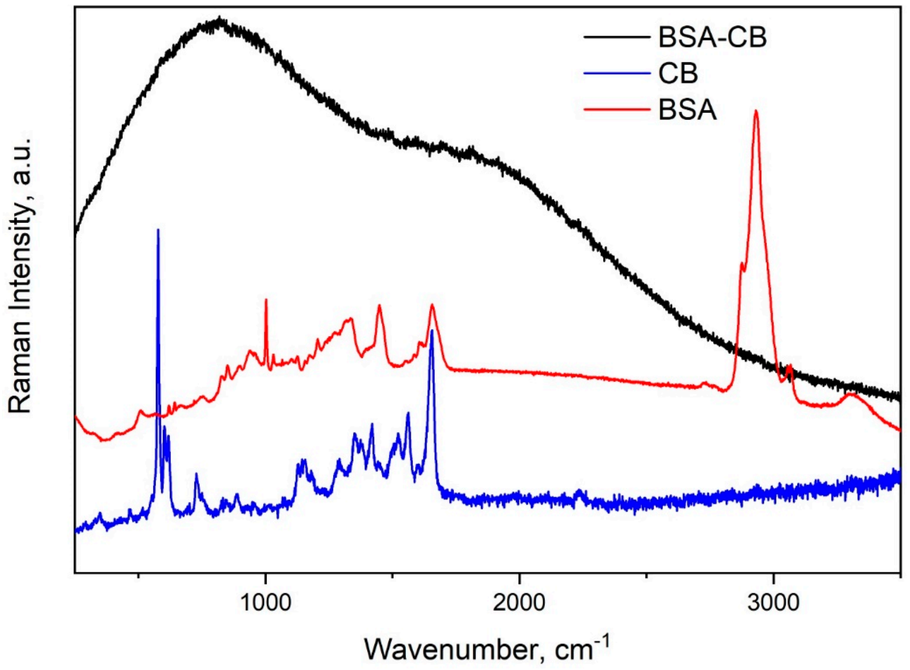
BSA-CB
CB
BSA
Raman Intensity, a.u.
1000
2000
3000
Wavenumber, cm-1

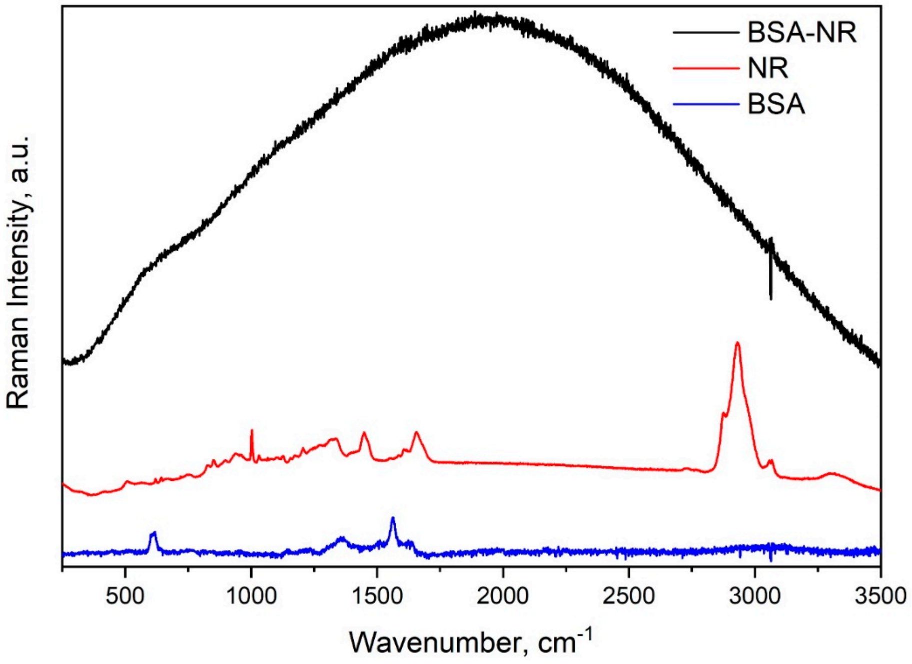
BSA-NR
NR
BSA
Raman Intensity, a.u.
500
1000
1500
2000
2500
3000
3500
Wavenumber, cm-1

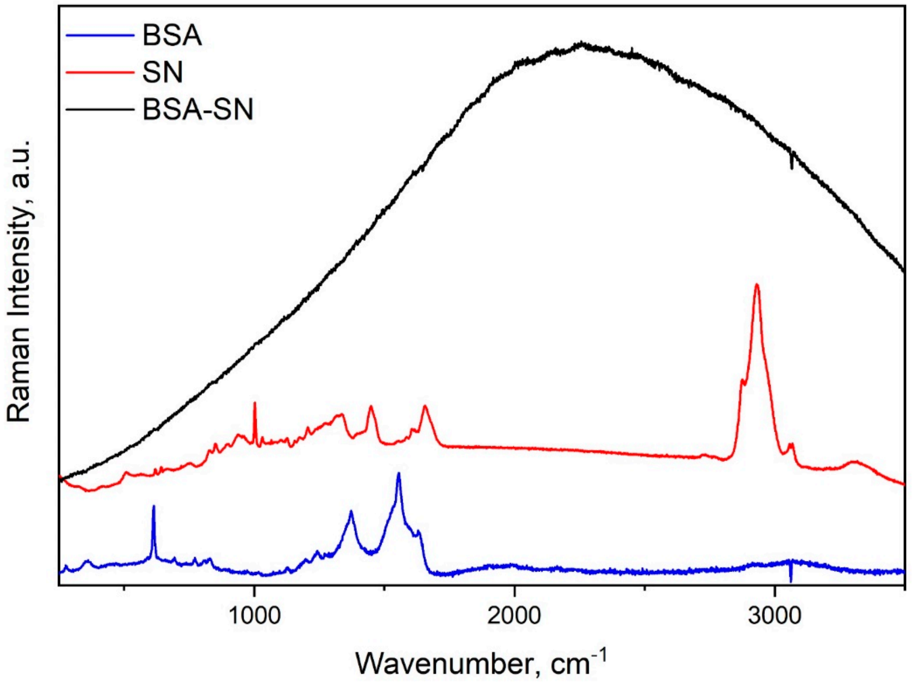

Figure S2. Raman spectra

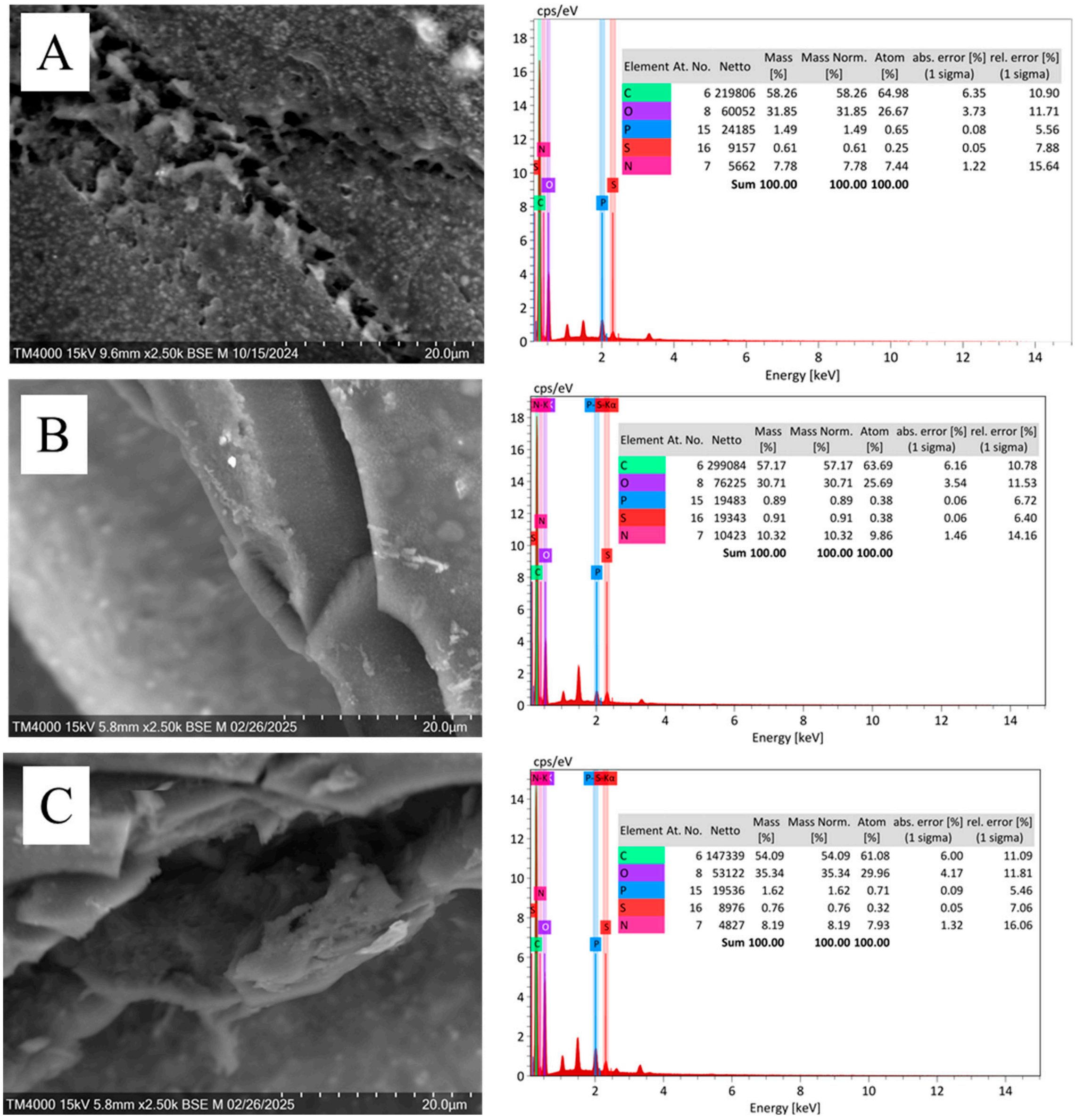

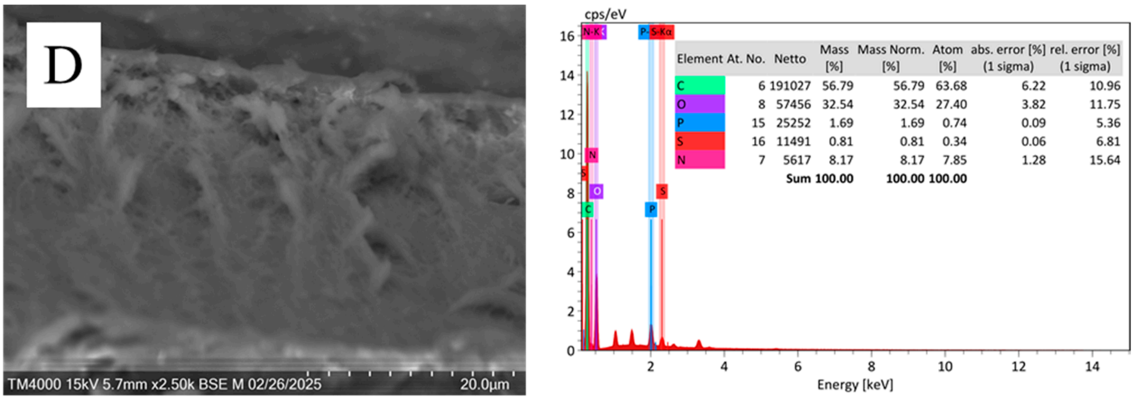
D
TM4000 15kV 5.7mm x2.50k BSE M 02/26/2025 20.0µm
cps/eV
Energy [keV]

Element	At. No.	Netto	Mass [%]	Mass Norm. [%]	Atom [%]	abs. error [%] (1 sigma)	rel. error [%] (1 sigma)
C	6	191027	56.79	56.79	63.68	6.22	10.96
O	8	57456	32.54	32.54	27.40	3.82	11.75
P	15	25252	1.69	1.69	0.74	0.09	5.36
S	16	11491	0.81	0.81	0.34	0.06	6.81
N	7	5617	8.17	8.17	7.85	1.28	15.64
		Sum	100.00	100.00	100.00		

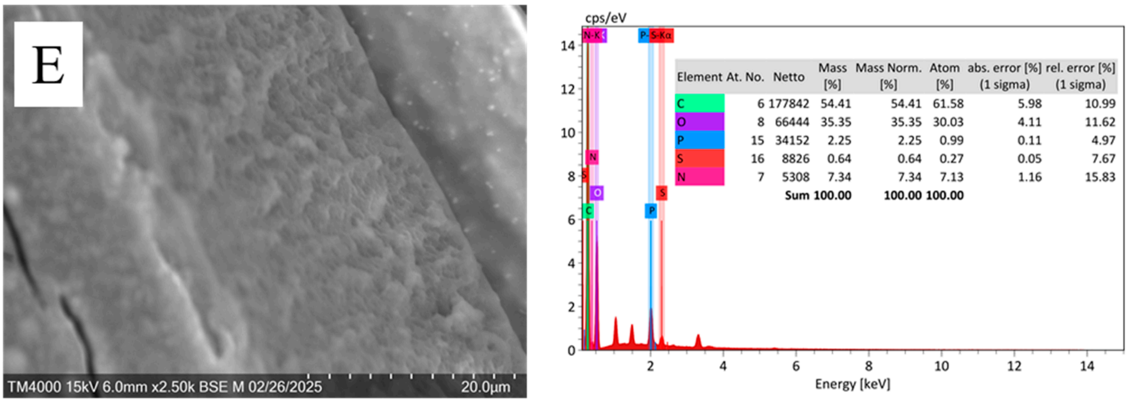
E
TM4000 15kV 6.0mm x2.50k BSE M 02/26/2025 20.0µm
cps/eV
Energy [keV]

Element	At. No.	Netto	Mass [%]	Mass Norm. [%]	Atom [%]	abs. error [%] (1 sigma)	rel. error [%] (1 sigma)
C	6	177842	54.41	54.41	61.58	5.98	10.99
O	8	66444	35.35	35.35	30.03	4.11	11.62
P	15	34152	2.25	2.25	0.99	0.11	4.97
S	16	8826	0.64	0.64	0.27	0.05	7.67
N	7	5308	7.34	7.34	7.13	1.16	15.83
		Sum	100.00	100.00	100.00		

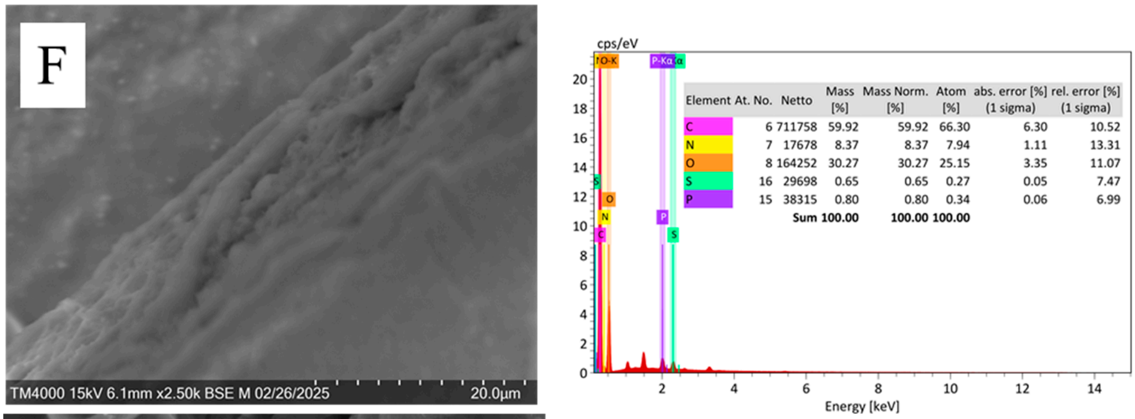
F
TM4000 15kV 6.1mm x2.50k BSE M 02/26/2025 20.0µm
cps/eV
Energy [keV]

Element	At. No.	Netto	Mass [%]	Mass Norm. [%]	Atom [%]	abs. error [%] (1 sigma)	rel. error [%] (1 sigma)
C	6	711758	59.92	59.92	66.30	6.30	10.52
N	7	17678	8.37	8.37	7.94	1.11	13.31
O	8	164252	30.27	30.27	25.15	3.35	11.07
S	16	29698	0.65	0.65	0.27	0.05	7.47
P	15	38315	0.80	0.80	0.34	0.06	6.99
		Sum	100.00	100.00	100.00		

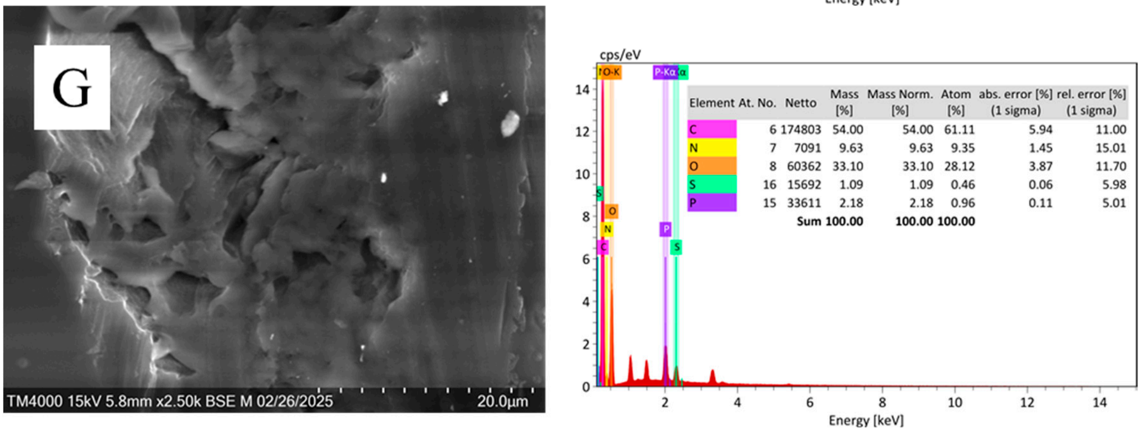
G
TM4000 15kV 5.8mm x2.50k BSE M 02/26/2025 20.0µm
cps/eV
Energy [keV]

Element	At. No.	Netto	Mass [%]	Mass Norm. [%]	Atom [%]	abs. error [%] (1 sigma)	rel. error [%] (1 sigma)
C	6	174803	54.00	54.00	61.11	5.94	11.00
N	7	7091	9.63	9.63	9.35	1.45	15.01
O	8	60362	33.10	33.10	28.12	3.87	11.70
S	16	15692	1.09	1.09	0.46	0.06	5.98
P	15	33611	2.18	2.18	0.96	0.11	5.01
		Sum	100.00	100.00	100.00		

Figure S3. SEM and EDX of the redox-active polymer: A. BSA-AA, B. BSA-CB, C. BSA-NR, D. BSA-NB, E. BSA-TH, F. BSA-TB, G. BSA-PHN.

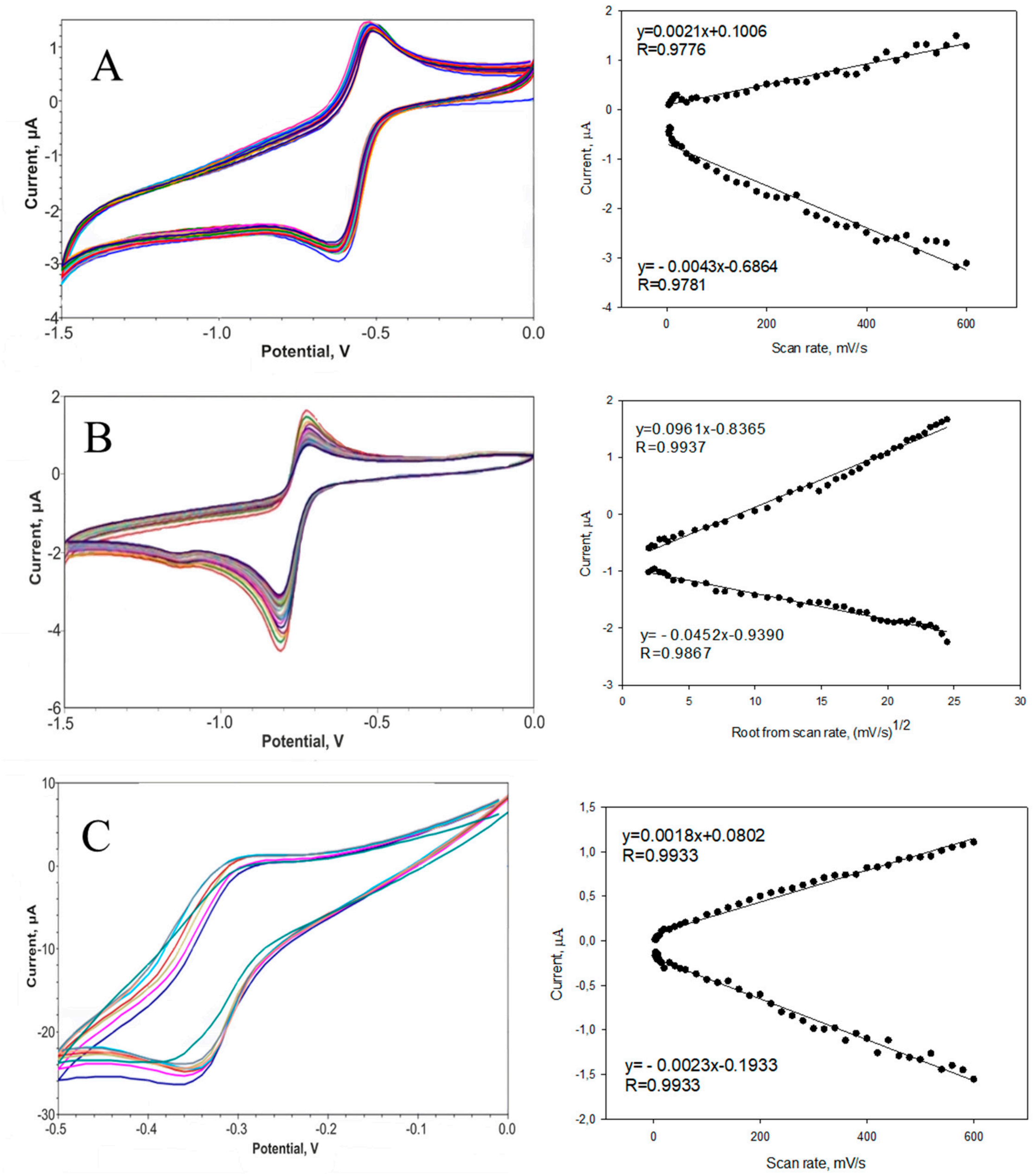

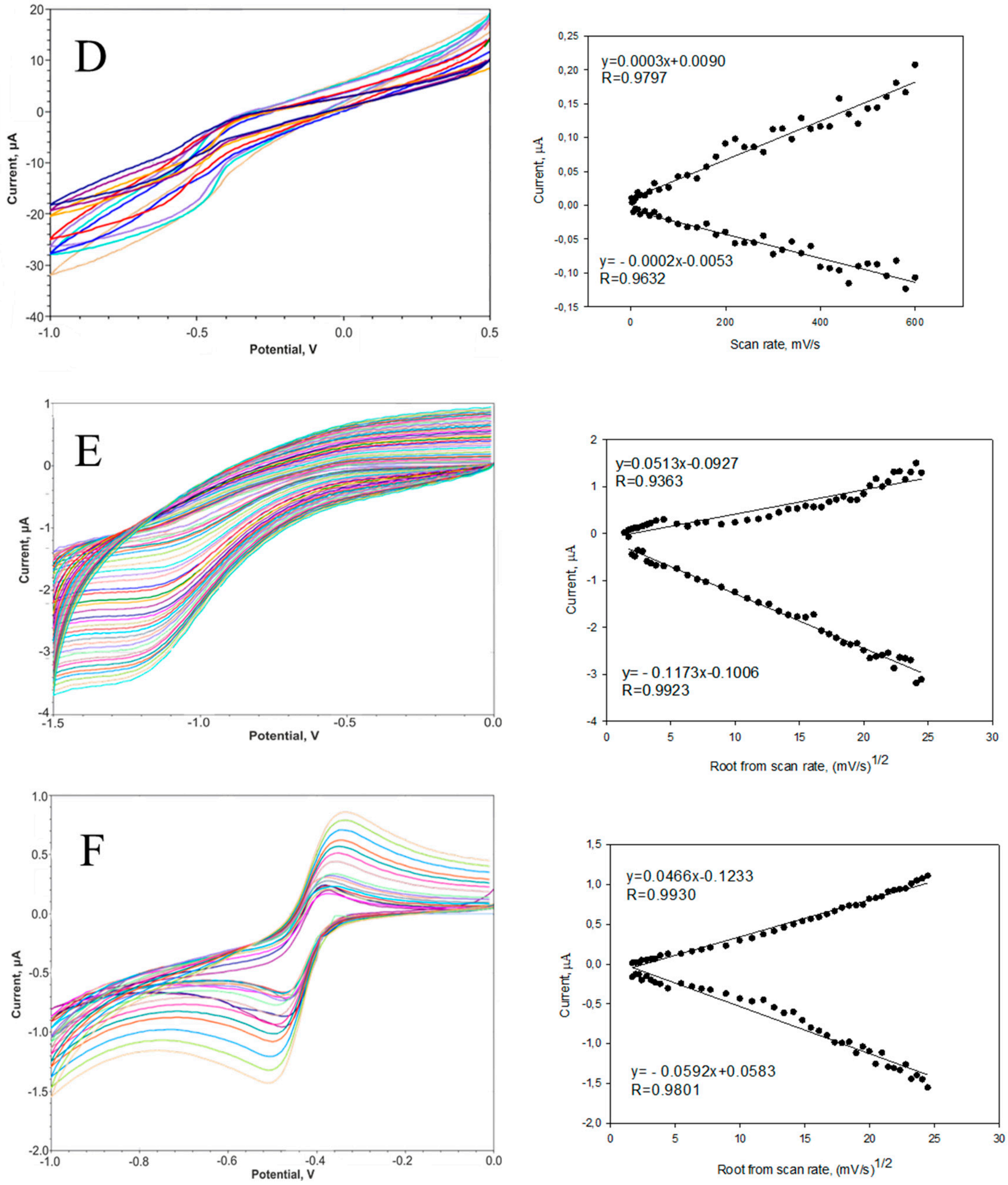

D
Current, µA
Potential, V
y=0.0003x+0.0090
R=0.9797
y= - 0.0002x-0.0053
R=0.9632
Current, µA
Scan rate, mV/s
E
Current, µA
Potential, V
y=0.0513x-0.0927
R=0.9363
y= - 0.1173x-0.1006
R=0.9923
Current, µA
Root from scan rate, (mV/s)^1/2
F
Current, µA
Potential, V
y=0.0466x-0.1233
R=0.9930
y= - 0.0592x+0.0583
R=0.9801
Current, µA
Root from scan rate, (mV/s)^1/2

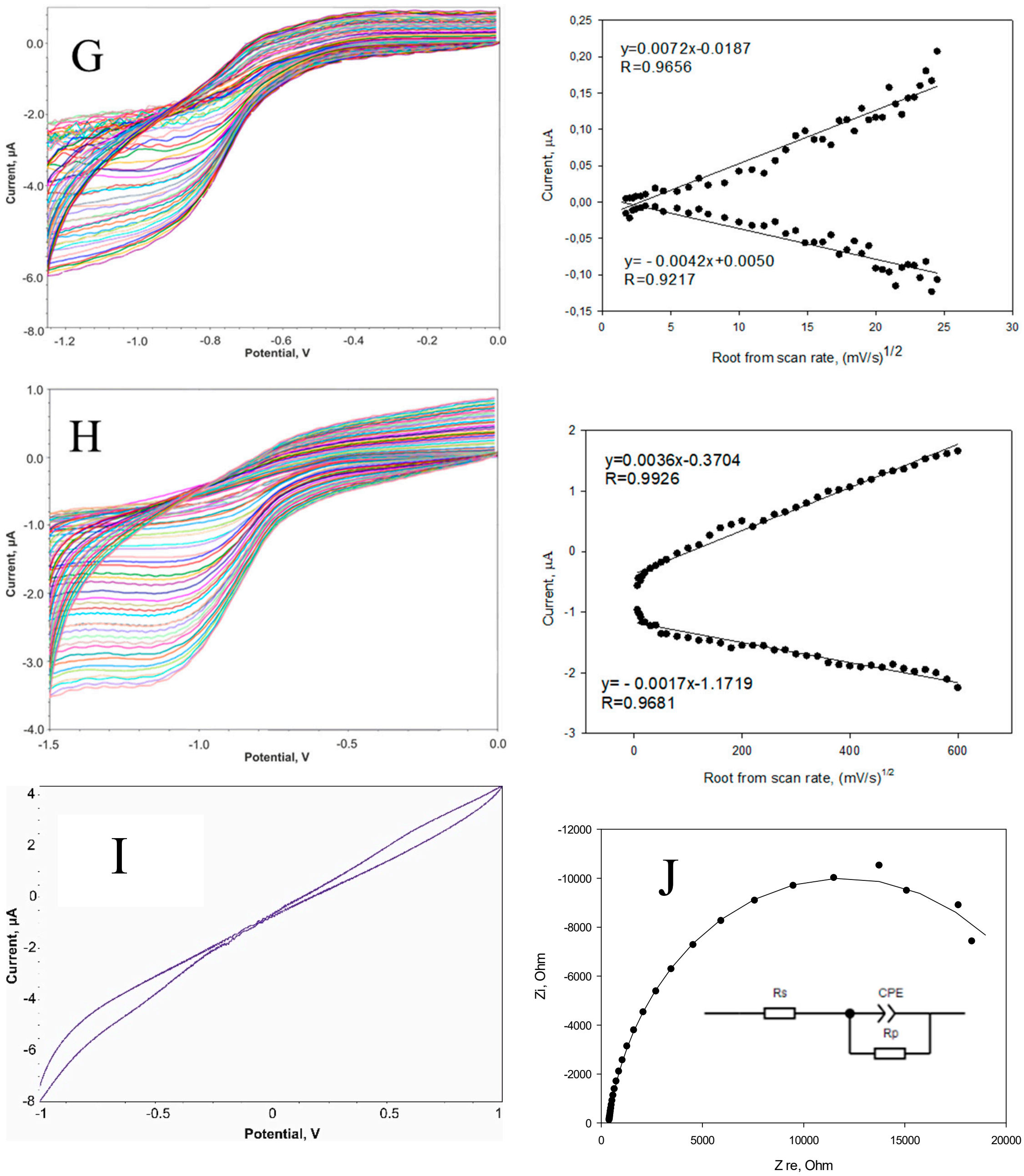

Figure S4. Electrochemical properties of polymers CV and heterogeneous constant rate calculation for: A. BSA-NB, B. BSA-NR, C. BSA-TH, D. BSA-CB, E. BSA-PHN, F. BSA-AA, G. BSA-SN, H. BSA-TB, I. BSA (Control), J. EIS of BSA (Control).

Text S1. Heterogeneous electron transfer constant rate determination

Three-electrode electrochemical cells are usually used to record voltammograms. The silver chloride electrode serves as a reference electrode, and the platinum electrode serves as an auxiliary electrode. The cyclic voltammetry method is used to calculate the heterogeneous electron transfer constant. In the case where the limiting stage is the surface reaction (Anode or Cathode current are proportional scan rate), the electron transfer process is described by the Laviron model. Based on the data obtained, a graph of the dependence of the limiting peak potential on the

logarithm of the scan speed is plotted. Using the graph, the electron transfer coefficient α and the heterogeneous electron transfer constant k are found using equation S1:

$$\log k = \alpha \log(1 - \alpha) + (1 - \alpha) \log \alpha - \log(RT/nFv) - \alpha(1 - \alpha)nF\Delta E/2{,}3RT \quad (S1)$$

where α is the transfer coefficient, R is the universal gas constant; T - temperature; n – number of electrons; F - Faraday number; ν - sweep speed; ΔE is the potential difference between the cathode and anodic peaks.

Text S2. The rate constant of interaction between the redox gel and enzyme determination

The change in the concentration of the mediator in the biocatalytic layer in the system "electrode – redox polymer – glucose oxidase – glucose" is described by the following equation (S2):

$$\frac{d[M]}{dx} = D\left(\frac{d^2[M]}{dx^2}\right) - \frac{k_{взаим}[E][M]}{1 + k_{взаим}\left(\frac{1}{k_2} + \frac{k_{-1} + k_2}{k_1 k_2 [S]}\right)[M]} \quad (S2)$$

where the first term is associated with the process of mass transfer of polymer redox sites (diffusion), and the second with their interaction with the enzyme. Under the condition of excess substrate and low concentrations of the mediator, equation (S2) is transformed to the form (S3):

$$\frac{d[M]}{dx} = D\left(\frac{d^2[M]}{dx^2}\right) - k_{взаим}[E][M] \quad (S3)$$

Under these conditions, the interaction constant of the mediator with the biomaterial can be calculated using the Nicholson and Shine equation (S4):

$$\frac{I_k}{I_d} = \sqrt{\frac{k_{int}[E]RT}{nFv}} \quad (S4)$$

where: I_k is the limiting current in the presence of a substrate, I_d is the limiting current in the absence of a substrate, k_{int} is the rate constant of interaction between the redox-cite and the biomaterial, [E] is the enzyme concentration, T is the temperature, R is the universal gas constant, ν is the scan rate, F is the Faraday constant.

To determine the rate constants, one can use the slope of the linear regression of the dependence of the ratio of the limiting anode currents in the presence and absence of the substrate (I_k/I_d) on $1/v^{1/2}$ according to formula (S5):

$$\tan \alpha = \sqrt{\frac{k_{int}[E]RT}{nF}} \quad (S5)$$

From it we can express the rate constant of interaction of the biomaterial with the mediator (S6)

$$k_{int} = \frac{(\tan\alpha)^2 nF}{[E]RT} \quad (S6)$$

Thus, to measure the interaction rate constant, it is necessary to record the CV in the presence and absence of the substrate at the same scan rate. When the substrate is added, an increase in the anodic peak current is observed. The dependences of the ratio of the anodic peak currents in the presence of excess substrate and without substrate on the square root of the sweep rate allow us to calculate the interaction constant using formula (S6).

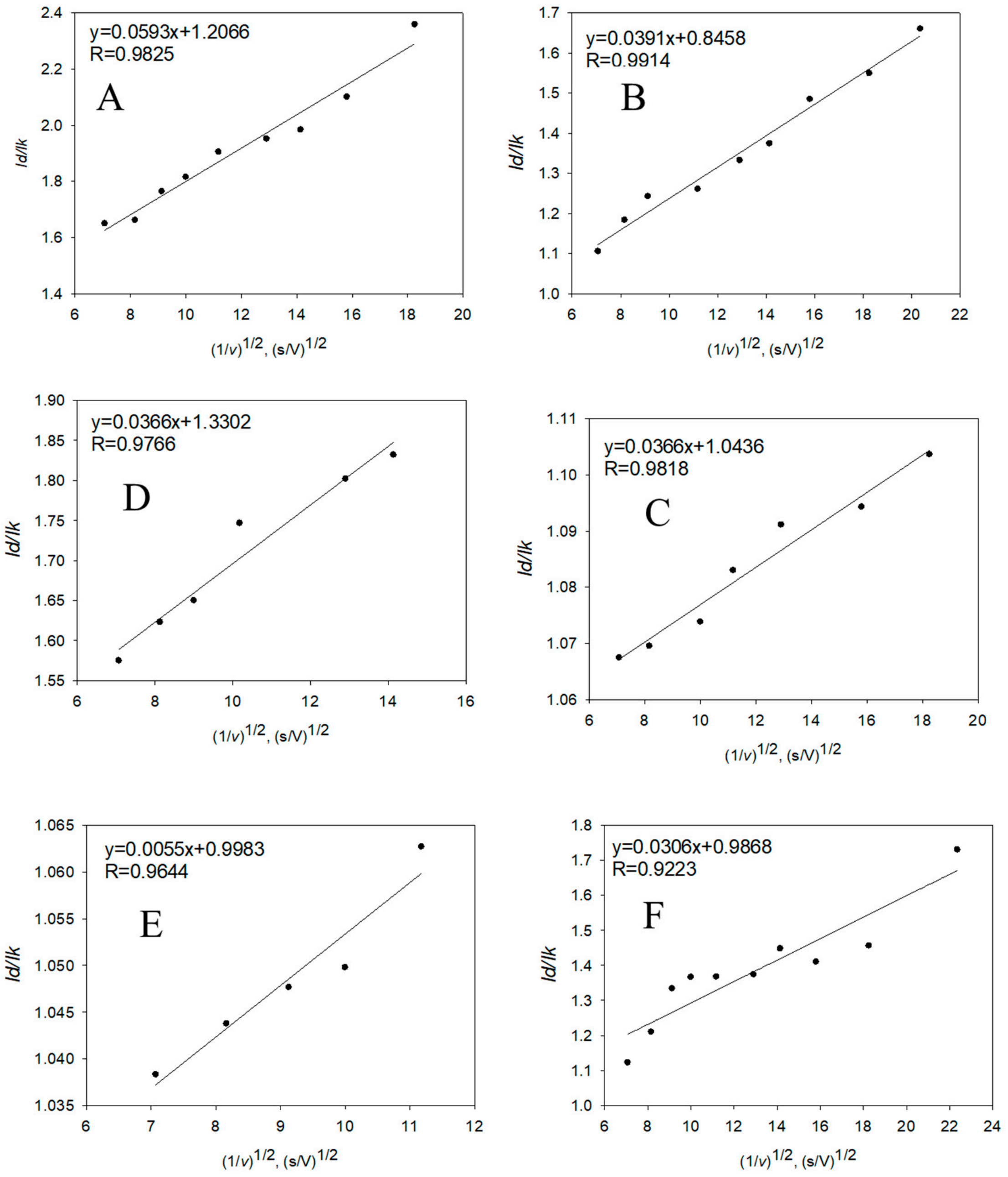

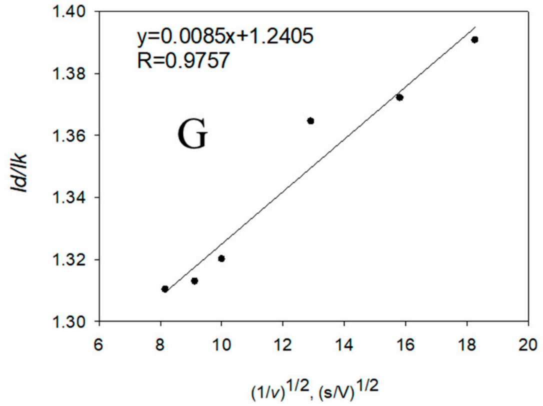

Figure S5. Dependence of (I_d/I_k) on $1/v^{1/2}$ for printed electrodes based on: A. BSA-NB;B. BSA-NR; C. BSA-TH; D. BSA-BC; E. BSA-SN; F. BSA-FHS; G. BSA-AA

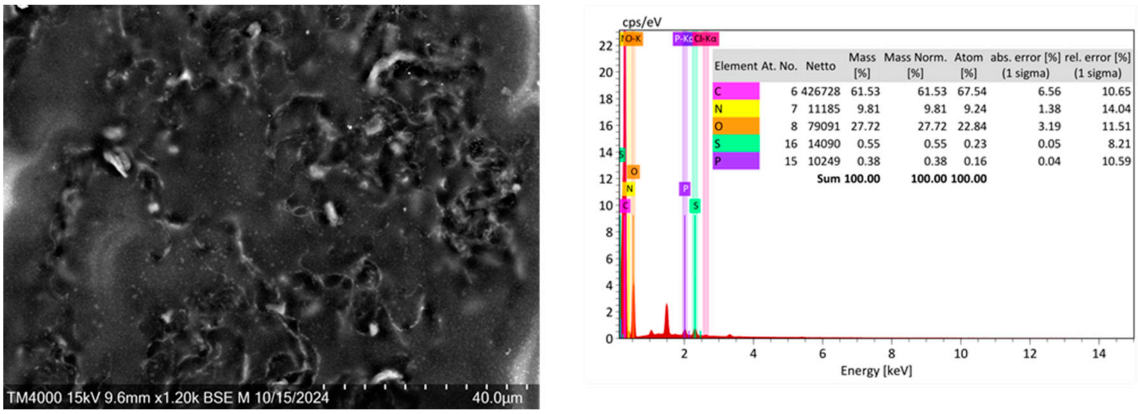

Figure S6. SEM and EDX of the BSA-NB-CNT composite.

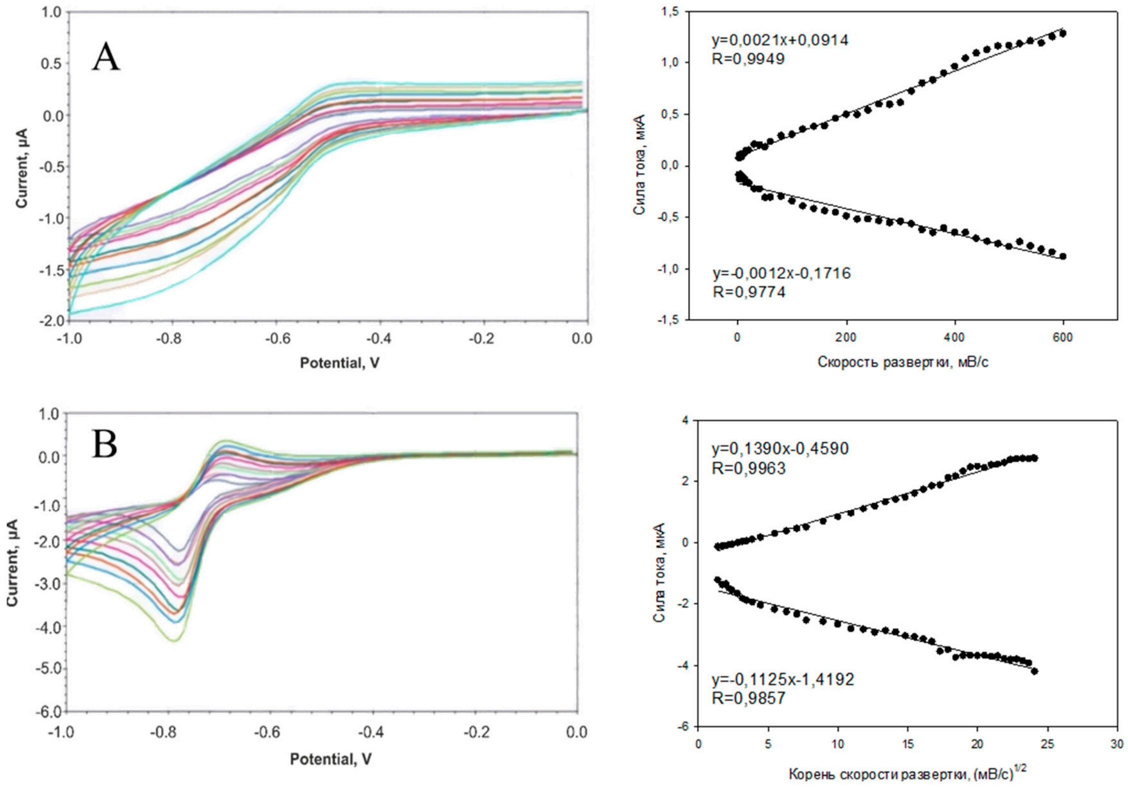

Figure S7. CV of composite for heterogeneous constant rate calculation A. BSA-NB-CNT composite. B. BSA-NR-CNT

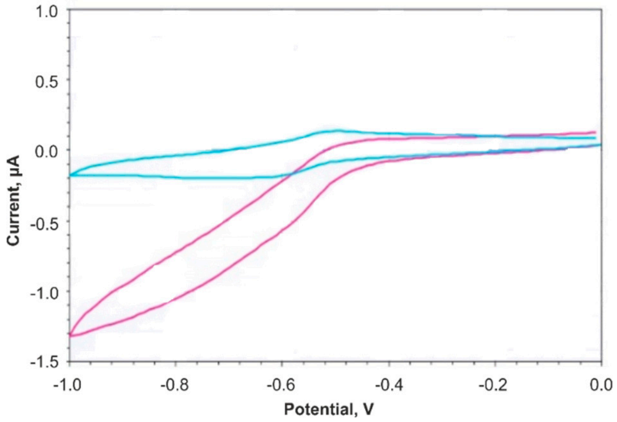

Figure S8. CV of the BSA-NB-CNT composite for constant rate calculation

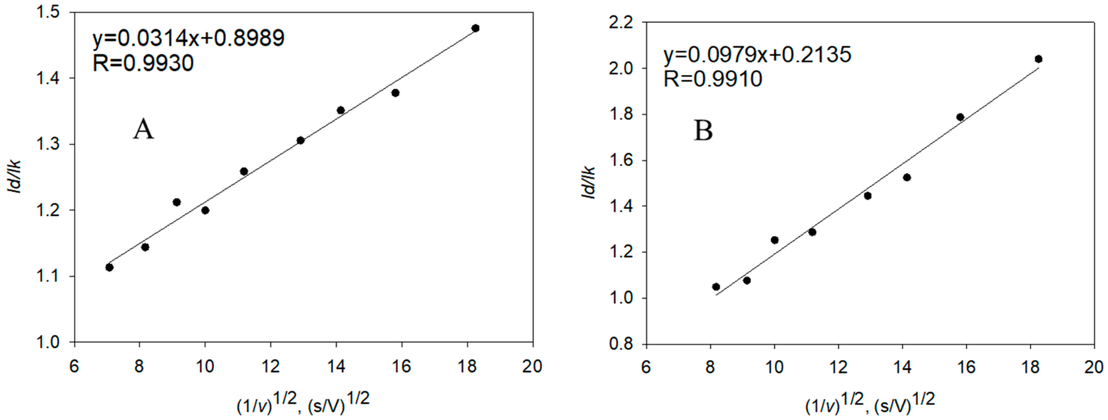

Figure S9. Dependence of (I_d/I_k) on $1/v^{1/2}$ for printed electrodes based on: A. BSA-NB-CNT; B. BSA-NR-CNT

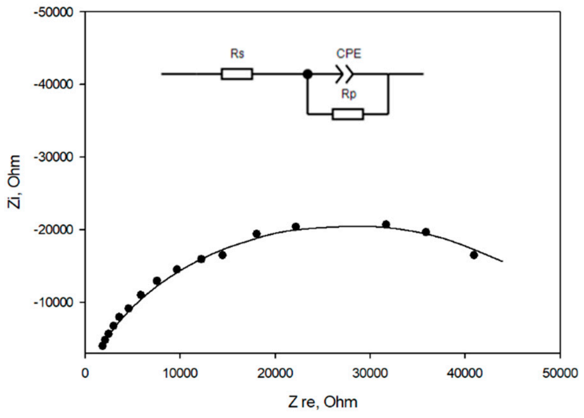

Figure S10. Electrochemical impedance spectra of *BSA-NB-CNT* composite and equivalent electrical circuit used for fitting the spectra

Table S1. The rate constant of interaction between the redox gel and enzymes

Composite	Enzyme	The rate constant of interaction between the redox gel and enzyme, L/mol·s
	AOx	2300 ± 700

BSA-NR-CNT	LOx	1820 ± 60
	Am+GOx	4000 ± 200

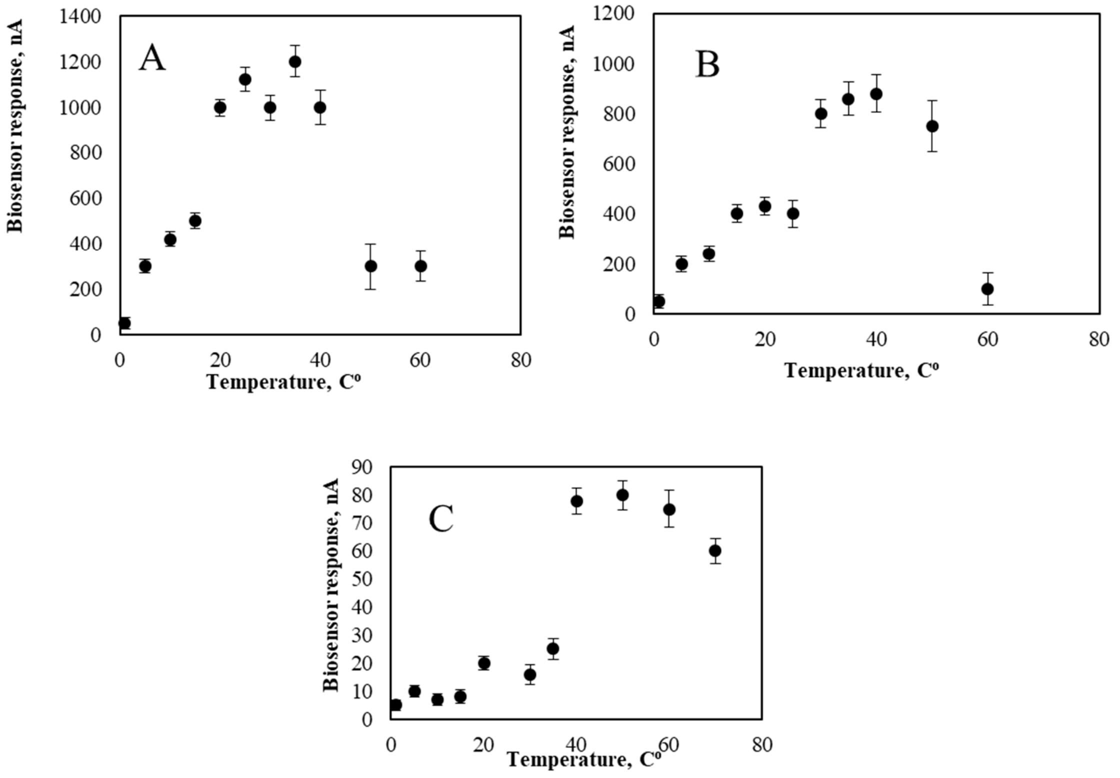

Figure S11. Effect of temperature on the response of the biosensor for the determination of: A. Ethanol; B. Lactate; C. Starch

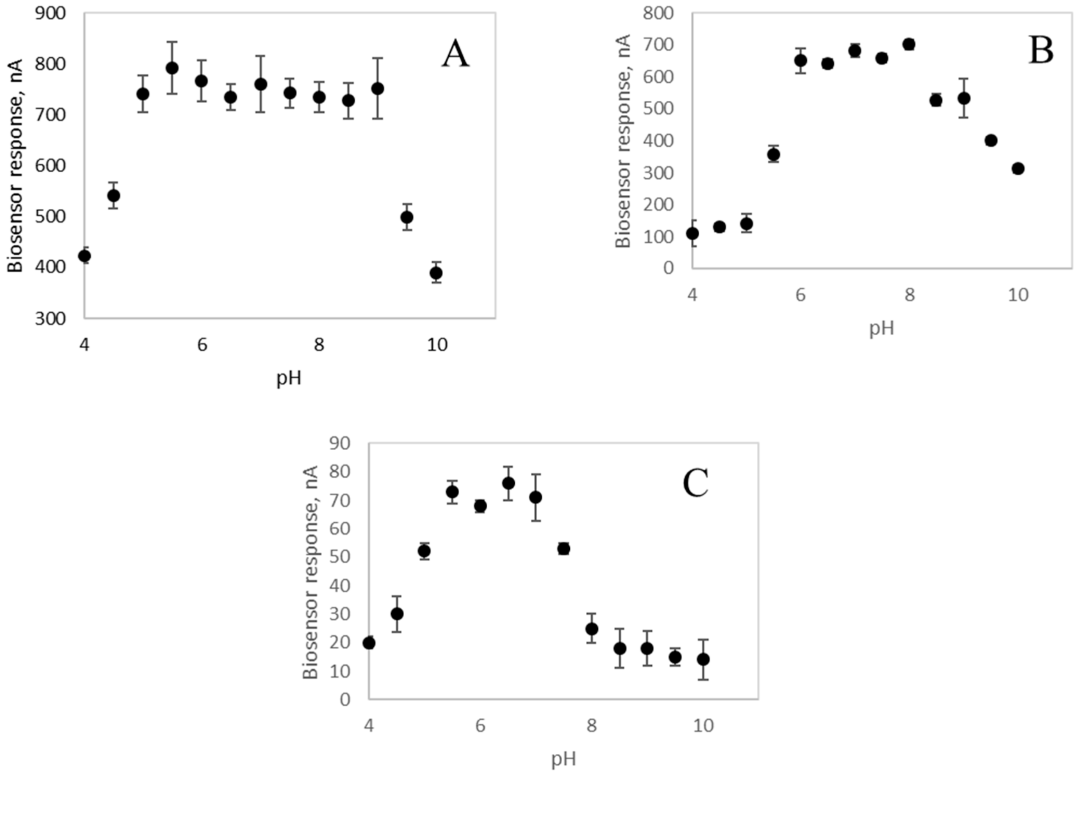

Figure S12. Effect of pH on the response of the biosensor for the determination of: A. Ethanol; B. Lactate; C. Starch

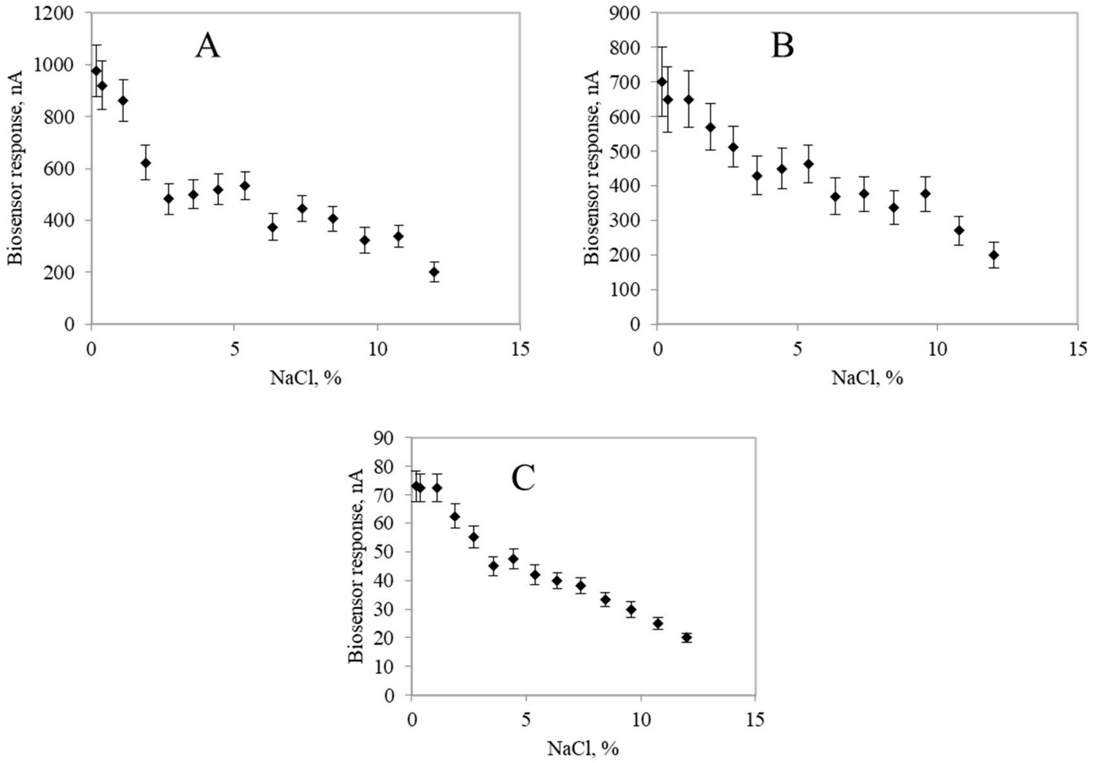

Figure S13. Effect of NaCl concentration on the response of the biosensor for the determination of: A. Ethanol; B. Lactate; C. Starch

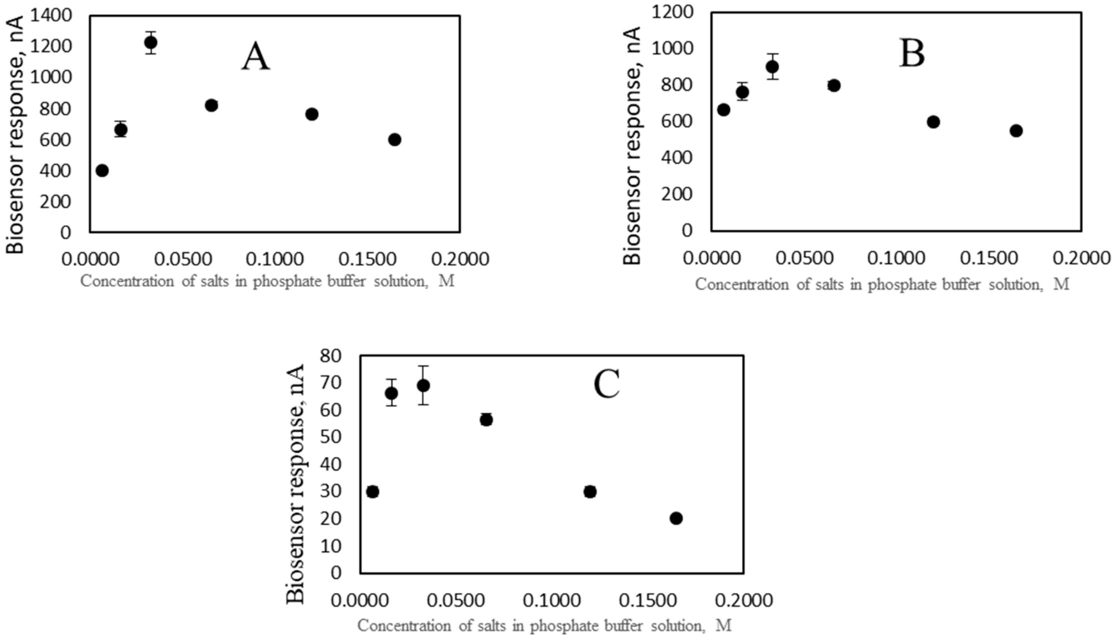

Figure S14. Effect of concentration of salts of phosphate buffer solution on the response of the biosensor for the determination of: A. Ethanol; B. Lactate; C. Starch

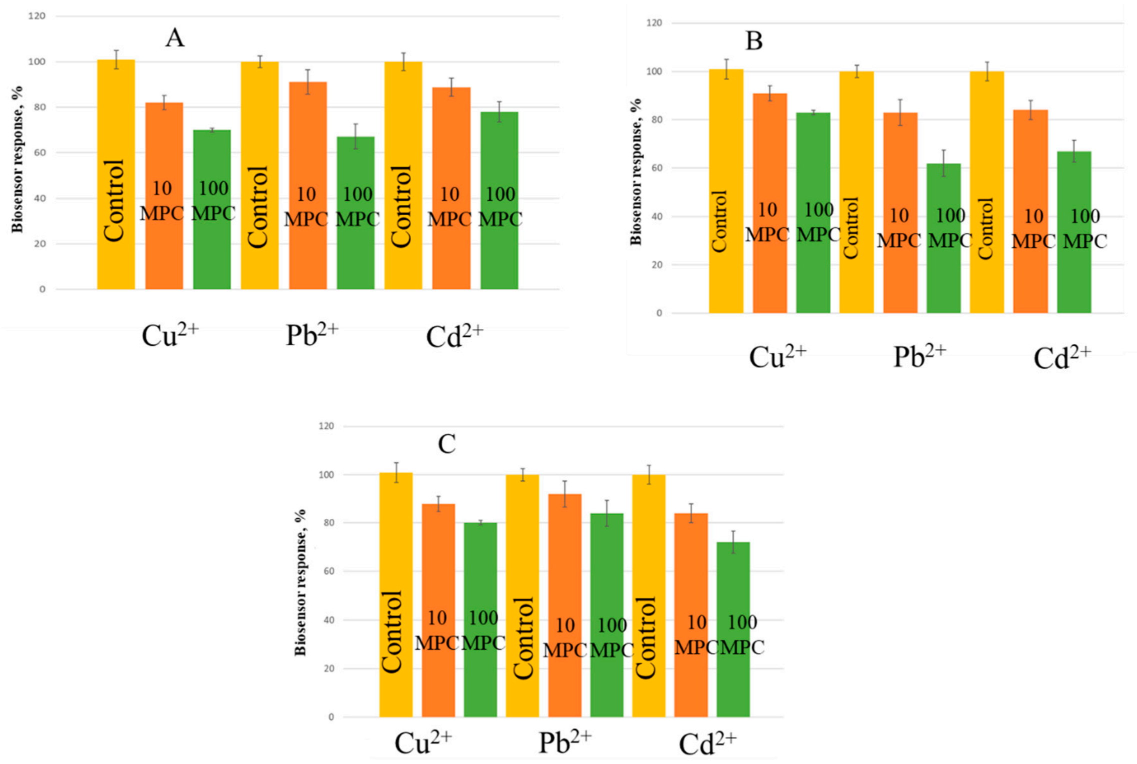

Figure S15. Effect of heavy metal ions on the response of the biosensor for the determination of: A. Ethanol; B. Lactate; C. Starch

Table S2. Results of measuring glucose, lactate, ethanol and starch using the developed biosensor and reference methods

Sample	Analyt	Concentration	
		Biosensor	Reference method
Wine 1	Ethanol	2.3±0.1 M	2.31±0.05 M
	Glucose	0.11 ±0.01 mM	0.116 ±0.003 mM
	Lactate	—	—
	Starch	—	—
Wine 2	Ethanol	2.1±0.1 M	2.15±0.05 M
	Glucose	0.16 ±0.01 mM	0.151 ±0.006 mM
	Lactate	—	—
	Starch	—	—
Wine 3	Ethanol	2.1±0.1 M	2.09±0.04 M
	Glucose	0.16 ±0.01 mM	0.151 ±0.006 mM
	Lactate	—	—
	Starch	—	—
Fermentation mass 1	Ethanol	—	—
	Glucose	0,20±0,02 M	0,23±0,02 M
	Lactate	—	—
	Starch	32 ± 1 g/L	31 ± 1 g/L
Fermentation mass 2	Ethanol	0.62±0.04 M	0.68±0.02 M
	Glucose	0.41±0.05 M	0.44±0.03 M

	Lactate	—	—
	Starch	0.2±0.04 g/L	0,2±0,05 g/L
Fermentation mass 3	Ethanol	1.8±0.1 M	1.72±0.03 M
	Glucose	0.004±0.001 M	0.004±0.001 M
	Lactate	—	—
	Starch	—	—
Kvass 1	Ethanol	0.09±0.01 M	0.09±0.01 M
	Glucose	—	—
	Lactate	19 ± 1 mM	20±4 mM
	Starch	—	—
Kvass 2	Ethanol	—	—
	Glucose	—	—
	Lactate	18 ± 1	17±2
	Starch	—	—
Kvass 3	Ethanol	0.04±0.01 M	0.041±0.004 M
	Glucose	—	—
	Lactate	20±2	22±4
	Starch	—	—
